# Discovery and characterization of novel inhibitors of the sodium-coupled citrate transporter (NaCT or SLC13A5)

**DOI:** 10.1038/srep17391

**Published:** 2015-12-01

**Authors:** Kim Huard, Janice Brown, Jessica C. Jones, Shawn Cabral, Kentaro Futatsugi, Matthew Gorgoglione, Adhiraj Lanba, Nicholas B. Vera, Yimin Zhu, Qingyun Yan, Yingjiang Zhou, Cecile Vernochet, Keith Riccardi, Angela Wolford, David Pirman, Mark Niosi, Gary Aspnes, Michael Herr, Nathan E. Genung, Thomas V. Magee, Daniel P. Uccello, Paula Loria, Li Di, James R. Gosset, David Hepworth, Timothy Rolph, Jeffrey A. Pfefferkorn, Derek M. Erion

**Affiliations:** 1 Worldwide Medicinal Chemistry, 610 Main street, Cambridge, MA 02139; 2 Pharmacokinetics, Dynamics, and Metabolism, Eastern Point road, Groton, CT 06340; 3 Cardiovascular, Metabolic & Endocrine Disease Research Unit, 610 Main street, Cambridge, MA 02139; 4 Worldwide Medicinal Chemistry, Eastern Point road, Groton, CT 06340; 5 Pharmacokinetics, Dynamics, and Metabolism, 610 Main street, Cambridge, MA 02139

## Abstract

Citrate is a key regulatory metabolic intermediate as it facilitates the integration of the glycolysis and lipid synthesis pathways. Inhibition of hepatic extracellular citrate uptake, by blocking the sodium-coupled citrate transporter (NaCT or SLC13A5), has been suggested as a potential therapeutic approach to treat metabolic disorders. NaCT transports citrate from the blood into the cell coupled to the transport of sodium ions. The studies herein report the identification and characterization of a novel small dicarboxylate molecule (compound 2) capable of selectively and potently inhibiting citrate transport through NaCT, both *in vitro* and *in vivo*. Binding and transport experiments indicate that **2** specifically binds NaCT in a competitive and stereosensitive manner, and is recognized as a substrate for transport by NaCT. The favorable pharmacokinetic properties of **2** permitted *in vivo* experiments to evaluate the effect of inhibiting hepatic citrate uptake on metabolic endpoints.

Fatty liver is a frequent co-morbidity of type 2 diabetes (T2D) and obesity. Therapies that could simultaneously target both pathogenic elevations in liver fat, and hyperglycemia, are highly desirable because in T2D, the elevations in circulating plasma glucose concentrations can be partially attributed to increased hepatic glucose production due to elevations in hepatic gluconeogenesis^1^,^2^,^3^. Additionally, increased liver fat associated with non-alcoholic fatty liver disease (NAFLD) is considered a pre-requisite for the development of non-alcoholic steatohepatitis (NASH)^4^. Thiazolidinediones (TZDs) used as anti-diabetes therapies exert multiple benefits on hepatic metabolism by reducing both liver fat and hepatic gluconeogenesis^5^,^6^. However, the side effects associated with TZDs such as weight gain and bone fractures have drastically reduced the use of this class of drugs^7^. More recently, glucagon-like peptide 1 (GLP-1) receptor agonists and dipeptidyl peptidase-4 (DPP-IV) inhibitors have become well established diabetes treatments with demonstrated benefits on reducing hepatic fat as well^8^. Alternative mechanisms capable of decreasing both hepatic lipid burden and glucose production remain of significant interest for the treatment of T2D.

Citrate is a key metabolite involved in intracellular signaling. Through allosteric modulation, citrate inhibits phosphofructokinase (PFK), thereby reducing glycolytic flux^9^. Citrate also promotes the polymerization and thus activation of acetyl-CoA carboxylase (ACC)^10^, which catalyzes the rate limiting step in *de novo* lipogenesis (DNL). Blocking the cellular uptake of citrate is hypothesized to have beneficial metabolic effects by reducing the energy burden placed on cells^11^. NaDC1, NaDC3, and NaCT (encoded by *SLC13A2*, *SLC13A3*, and *SLC13A5*, respectively) are critical carrier proteins that co-transport sodium ions with Krebs cycle intermediates such as citrate and succinate from the extracellular space into the cell^12^. In humans, NaDC1 and NaDC3 are di- and tri-carboxylate transporters primarily expressed in the intestine and kidney. While these transporters bind citrate with a lower affinity than other Krebs cycle intermediates, NaDC1 and NaDC3 play an important role in the absorption and excretion of citrate^13^. *SLC13A5* expression is enriched in the human liver and appears to be the predominant plasma membrane citrate transporter expressed^13^. NaCT, on the other hand, is the only known plasma membrane carrier to preferentially transport citrate over dicarboxylates^14^. The expression profile and reported substrate selectivity of NaCT make it an attractive target to alter hepatic citrate uptake^15^.

The regulation of metabolic processes by *SLC13A5* was revealed through studies with its homolog in *Drosophila melanogaster* and *Caenorhabditis elegans*^16^,^17^,^18^. In these species, inactivation of *SLC13a5* specifically resulted in lifespan extension, analogous to the results observed with caloric restriction. In a mammalian model, *SLC13a5* knockout (KO) mice show improvements in glycemic control as demonstrated by increases in the glucose infusion rate required to maintain euglycemia in a hyperinsulinemic-euglycemic clamp, which can be attributed to suppression of glucose production^19^. Additionally, *SLC13a5* KO mice that have been fed a high fat diet (HFD) display reductions in body weight and hepatic lipid concentrations including diacylglycerides (DAG) and triglycerides (TAG) relative to their wild type (WT) counterparts. Studies using antisense oligonucleotides (ASO) to knock down *SLC13a5* in rats on a HFD corroborated the KO data, demonstrating improvements in insulin responsiveness which was attributed to improvements in hepatic glucose production and insulin responsiveness^20^. Taken together, these data suggest that pharmacological inhibition of NaCT may prove to be a beneficial strategy for treating metabolic disorders.

Sun *et al.* (2010) reported small molecule inhibitors of NaCT that were identified via virtual docking using a homology model of NaCT, and a proteoliposome-based assay was used to measure their weak inhibitory activity on citrate transport (<73% inhibition at 1 mM)^21^. NaDC1 and NaDC3 inhibitors reported by Pajor and Randolph (2007) also displayed weak inhibition of NaCT in transfected CUBS cells^22^. However, in our hands these compounds exhibited cytotoxicity in HEK-293-derived cell-based assays (using a CellTiter-glo® assessment) thereby confounding the interpretation of citrate uptake activity (Figure S1). Moreover, all previously reported NaCT inhibitors displayed poor ADME properties precluding their use in *in vivo* experiments. More recently, Colas and collaborators described the identification of new NaDC1 and NaDC3 inhibitors via virtual docking in homology models, with one example also displaying weak inhibitory activity against NaCT (~30% inhibition at 500 μM)^23^. Herein, the identification of the first potent and selective small molecule probe for NaCT which inhibits cellular citrate uptake *in vitro* and hepatic citrate uptake *in vivo* is described. Inhibition of NaCT resulted in lower hepatic lipid concentrations and improved glycemic control in mice fed a HFD, which supports the further exploration of NaCT inhibitors for the treatment of metabolic diseases.

## Results

### Identification and characterization of dicarboxylate 2 as an inhibitor of NaCT-mediated citrate uptake

**Figure 1:**
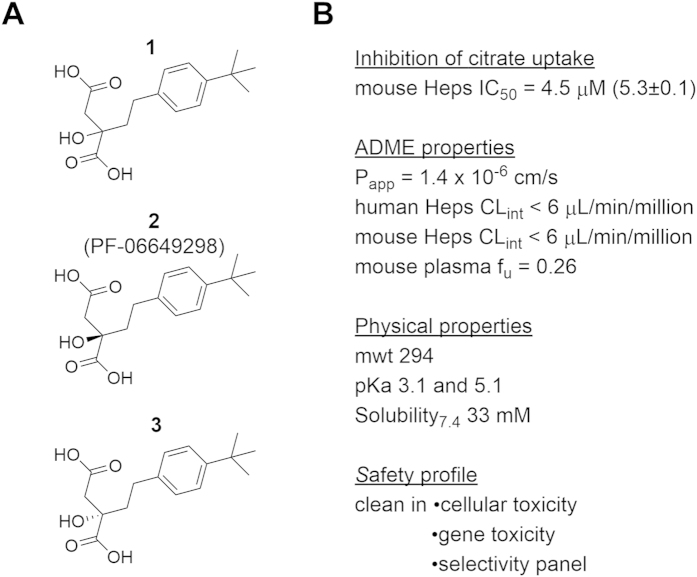
Structure of dicarboxylates 1–3 and *in vitro* profile of dicarboxylate 2 (PF-06649298). (**A**) Chemical structure of racemic dicarboxylate **1** and its enantiomers **2** and **3**. (**B**) *In vitro* profile of dicarboxylate **2**. Inhibition of citrate uptake was measured in mouse hepatocytes. IC_50_ is reported as a geometric mean of at least 3 replicates with pIC_50_ ± SD in parentheses. mwt = molecular weight. P_app_ = apparent passive permeability. Heps = hepatocytes. CL_int_ = intrinsic clearance. f_u_ = fraction unbound.

To identify NaCT inhibitors, a virtual search of Pfizer’s compound library was conducted based on structural similarities to the transporter’s preferred substrate citrate. 500 compounds were selected for testing in a HEK-293-derived stable cell line overexpressing *SLC13A5* (HEK_NaCT_) to measure their effect on cellular citrate uptake. This effort led to the identification of racemic dicarboxylate **1** (Fig. 1A) which inhibited 50% of the citrate transport from the media into the cells at a concentration of 0.80 μM (Table 1). No cellular toxicity was observed under these conditions using a CellTiter-glo^®^ assessment.

Racemic compound **1** was resynthesized according to the sequence depicted in Scheme S1. Ketoester **6** was accessed via a copper mediated coupling of ethyl chlorooxoacetate and *t*-butyl ethynylbenzene. A Mukaiyama aldol reaction of **6** with silyl enol ether **7** afforded diester **8**. Alkyne reduction followed by chiral separation and hydrolysis provided single enantiomers **2** and **3** with respective IC_50_ values of 0.41 μM and >25 μM in HEK_NaCT_ cell line (Fig. 1A and Table 1). The stereochemical configuration of **2** was confirmed as *R* through single crystal X-ray analysis of the inactive enantiomer **3**, which was determined to have the *S* configuration (Figure S2). To assess the inhibitory activity of **2** in a cell line in which the transporter was in its native environment, we used cryopreserved human hepatocytes and confirmed inhibition although with a significant shift in potency (IC_50_ = 16.2 μM, Table 1). Compound **2** was also active in cryopreserved mouse hepatocytes with an IC_50_ of 4.5 μM (Fig. 1B).

Additional profiling of **2** demonstrated selectivity within the SLC13 transporter family. No measurable inhibition of citrate or succinate uptake was detected in HEK-293-derived systems overexpressing NaDC1 and NaDC3 (Table 1). In addition, no significant interaction/inhibition was observed with ATP-citrate lyase, the hERG channel, major human CYP450s (CYP1A2, CYP2C8, CYP2C9, CYP2D6, CYP3A4), and over 65 other individual targets including various transporters, ion channels, receptors and enzymes (Table S1). Overall, a clean *in vitro* safety profile was obtained for **2** with negative results in genetic toxicity assays and no observable toxicity in HepG2 cells at concentrations up to 300 μM and 72 hours incubation. Compound **2** is a low molecular weight and polar dicarboxylate with pKa values of 3.1 and 5.1, conferring excellent aqueous solubility, high metabolic stability (clearance from hepatocytes could not be detected) and low passive membrane permeability (P_app_, as assessed *in vitro* with MDCK-LE cell monolayer, Fig. 1B).

### Dicarboxylate 2 is both a substrate and inhibitor of NaCT

**Figure 2:**
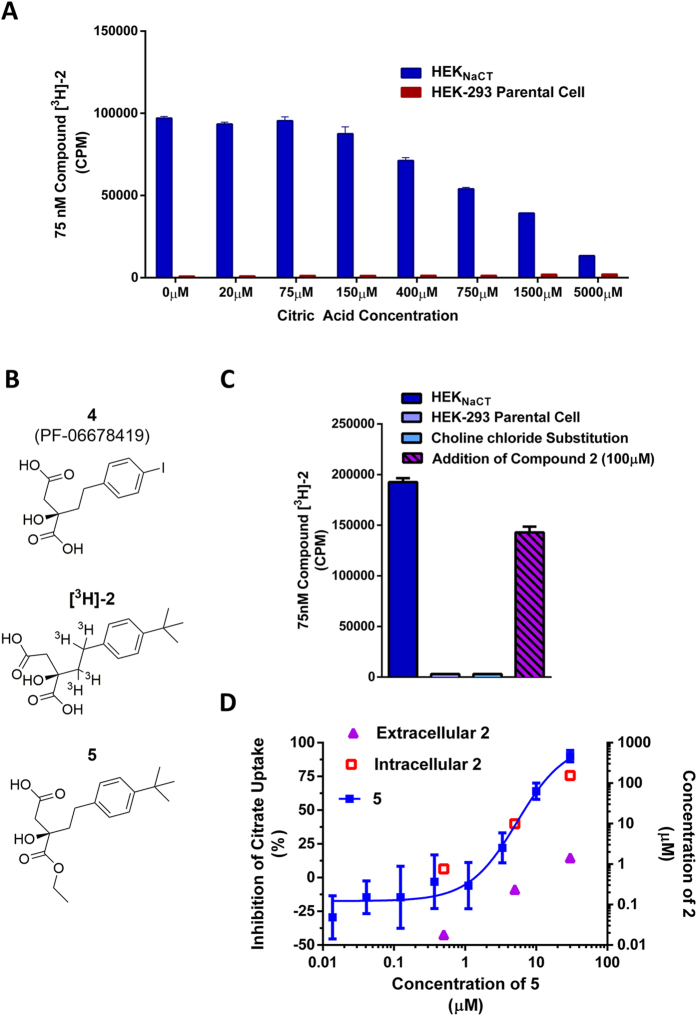
Characterization of the interaction between compound 2 and HEK_NaCT_ cells. (**A**) Interaction of compound [^**3**^**H]-2** (75 nM) with HEK-293 and HEK_NaCT_ cells competed with increasing amounts of citric acid (n = 3). (**B**) Chemical structure of dicarboxylate **4**, tritiated [^**3**^**H]-2** and ester **5**. (**C**) Using compound [^**3**^**H]-2** (75 nM) from left to right, interaction after 2 hours of incubation in; (1) HEK_NaCT_, (2) parental HEK-293 cells, (3) HEK_NaCT_ with sodium chloride replaced by choline chloride, and (4) following an additional 2 hours incubation with 100 μM of compound **2** (n = 3). (**D**) Inhibition of citrate uptake in human hepatocytes using the prodrug **5** with measurements of intracellular and extracellular conversion to compound **2** (n = 5).

In order to investigate the mode of action by which **2** inhibits the cellular transport of citrate, a radiolabeled analog was required. With this goal in mind, the iodo-substituted compound **4** (PF-06678419) was synthesized via a Sonogashira reaction with 1,4-diiodobenzene and terminal alkyne **12**. The resulting alkyne **13** was reduced with *p*-tolylhydrazide followed by chiral separation and hydrolysis to afford diacid **4** (Fig. 2B and Scheme S2). Compound **4** showed inhibition of citrate uptake in the HEK_NaCT_ cells with potency similar to that of **2** (IC_50_ = 0.44 μM). However, we did not pursue the preparation of a radiolabeled analog with compound **4** as it did not show complete selectivity for NaCT over NaDC1 and NaDC3 (Table 1). On the other hand, suitable specific activity was obtained with a tritiated version of **2** ([^**3**^**H]-2**, Fig. 2B). This compound was prepared by alkyne reduction of the chiral diacid **16** with palladium on carbon and tritium gas (Scheme S3), and evaluated in whole cell experiments where radioactivity was measured. An interaction between [^**3**^**H]-2** and the NaCT-expressing cells was observed in whole cell experiments and this binding could be out-competed with increasing concentrations of citrate (Fig. 2A). The competitive nature of this interaction with citrate suggested a common binding site for **2** and citrate on NaCT. As opposed to the HEK_NaCT_ cells, no significant interaction was observed with the parental HEK-293 cells (Fig. 2A,C). Since NaCT expression is not detected in qRT-PCR analysis of the parental HEK-293 cell (data not shown), these data support a specific interaction between [^**3**^**H]-2** and NaCT. The presence of sodium in the cell media was required for this interaction as the entire signal observed with the HEK_NaCT_ cells was abolished when sodium chloride was replaced with choline chloride (Fig. 2C). Transport of citrate via NaCT was previously shown to be a sodium-dependent process as well^24^. When HEK_NaCT_ cells were incubated with a 75 nM solution of [^**3**^**H]-2**, washed with buffered saline and the media replaced with 100 μM of **2** for 2 hours, a large fraction of the total radioactive signal remained (Fig. 2C). This observation could be attributed to a slow dissociation or irreversible interaction; however, based on the overall data generated in these experiments, as well as the structural similarity of **2** with citrate, it was hypothesized to be the result of NaCT-mediated cellular uptake of the dicarboxylate.

The data supporting active transport of **2** by NaCT prompted investigation of the intra- and extra-cellular concentrations of compound in the cell based assays. After a 1 hour incubation, compound **2** was not detected at significant levels inside the HEK-293 parental cells which do not express *SLC13A5* (data not shown). In contrast, incubation with the NaCT-expressing cells resulted in asymmetric distribution of **2** into the intracellular compartment. The measured ratio of intracellular to media unbound compound concentrations (K_puu_) was 29 in the HEK_NaCT_ cells and 4.8 in human hepatocytes (Table 1). The inactive enantiomer **3** did not reach substantial intracellular concentrations in HEK_NaCT_ or in human hepatocytes, suggesting **3** is not a substrate for the transporter (Table 1). These data are consistent with the hypothesis that NaCT actively transports **2** into cells and that the interaction is stereosensitive.

Since the active transport of **2** by NaCT generates unequal intra- and extracellular concentrations in the cell-based assays, a potential ester pro-drug **5** was prepared (Fig. 2B and Scheme S4) with the aim of studying the inhibition event and its location. This compound, in which one of the carboxylates had been masked as an ethyl ester, showed a high degree of passive permeability (P_app_ = 13 × 10^−6^ cm/sec) relative to the parent dicarboxylate **2**. Further, while ester **5** was stable in assay media (t_1/2_ > 120 min) and HEK_NaCT_ cells (data not shown), it had a short half-life of 29 minutes in metabolically competent human hepatocytes. These results supported the potential of **5** to deliver dicarboxylate **2** into the intracellular compartment. Consistent with this, **5** showed no effect on the uptake of citrate in the HEK_NaCT_ cells (IC_50_ >25 μM) but in cryopreserved human hepatocytes, citrate uptake was inhibited with an IC_50_ of 6.76 μM (Table 1, Fig. 2D, blue curve). The intracellular unbound concentration of dicarboxylate **2** derived from esterase mediated hydrolysis of prodrug **5** in human hepatocytes was measured at standardized time points with three different extracellular concentrations and was found to correlate with inhibition of citrate uptake (Fig. 2D, red squares). Due to the high stability of the prodrug in the assay media and restricted permeability of the parent diacid **2**, concentrations of **2** observed in the media were well below the IC_50_ for inhibition of NaCT (Fig. 2D, purple triangles) and not sufficient to account for the observed effect since the transport of citrate was not affected in human hepatocytes when treated with similar concentrations of **2** (Figure S3). These results indicate that the intracellular accumulation of dicarboxylate **2**, delivered by active NaCT-mediated transport or by hydrolysis of ester prodrug **5**, inhibits the transport of citrate. The contribution of the extracellular **2** to inhibition of citrate transport could not be evaluated due to its active cellular uptake.

### Dicarboxylate 2 modulates citrate metabolism in human hepatocytes

**Figure 3:**
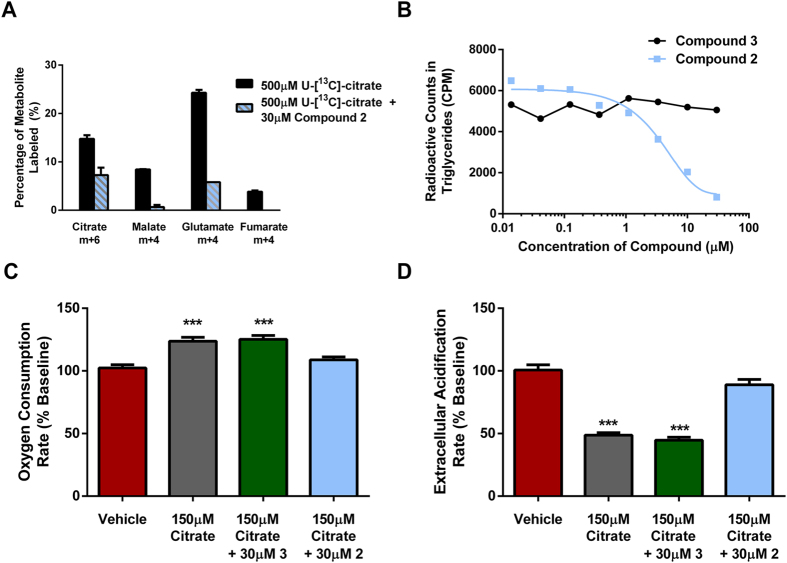
Impact of citrate and compound 2 or 3 on cellular metabolism. (**A**) U-[^13^C]-citrate uptake and incorporation into malate, glutamate, and fumarate as a percentage of total cellular concentrations. No label was detected in cells treated with unlabeled citrate (n = 2). (**B**) Incorporation of [^14^C]-citric acid into triglycerides in human hepatocytes, treated with either compound **2** or **3** (n = 1). (**C,D**) Oxygen consumption and extracellular acidification rates in HEK_NaCT_ treated with vehicle, compound **2** or **3** (***P < 0.001; one-way ANOVA - Tukey’s multiple comparison test).

In order to better understand the *in vitro* metabolic fate of citrate, human hepatocytes were incubated with 500 μM of uniformly labeled citrate (U-[^13^C]-citrate). Substantial uniform labeling of citrate, malate, glutamate, and fumarate was observed in cells treated with the labeled citrate, which was reduced in the presence of 30 μM compound **2** (Fig. 3A). In human hepatocytes treated with radiolabeled citrate for 24 hours, label incorporation in TAG decreased dose-dependently with **2** but not with inactive enantiomer **3** (Fig. 3B). To assess the roles of citrate in cellular metabolism and the consequences of limiting citrate uptake through inhibition of the NaCT transporter, HEK_NaCT_ cells were incubated with 150 μM citrate and either vehicle, **2** or **3**, and results were recorded using a Seahorse® apparatus. The presence of citrate alone acutely increased cellular oxygen consumption rate (OCR), while 30 μM of compound **2**, but not **3**, prevented citrate induced increase in mitochondrial respiration (Fig. 3C). Since citrate inhibits PFK, a key regulator of glycolytic flux, the extracellular acidification rate (ECAR) was measured as a marker of glycolytic flux. Treatment of HEK_NaCT_ cells with citrate acutely suppressed ECAR by 50% which was restored by 30 μM of compound **2** but not by **3** (Fig. 3D). These data support a critical role for citrate in glycolysis and mitochondrial metabolism.

### Dicarboxylate 2 blocks *in vivo* hepatic uptake of citrate in mice, reduces plasma glucose concentration and reverses glucose intolerance in mice fed a HFD

**Figure 4:**
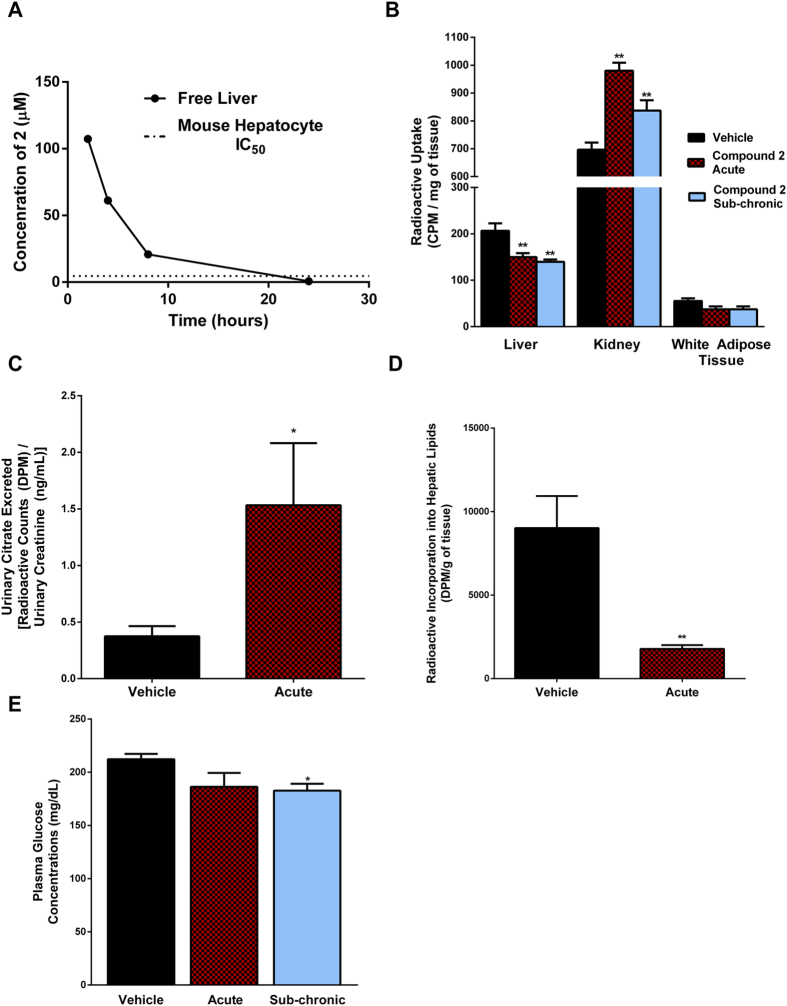
*In vivo* citrate metabolism in mice treated with compound 2. (**A**) Free liver exposure of compound **2** in mice after an oral dose of 250 mg/kg. (**B**) Uptake of radioactive [^14^C]-citric acid in liver, kidney and white adipose tissue in DIO mice treated with either vehicle, a single 250 mg/kg dose (‘acute’) of compound **2**, or 3 days 250 mg/kg BID (‘sub-chronic’) of compound **2** (**P<0.01, One-way ANOVA – Dunnett’s post hoc test, n = 9–10). (**C**) Radioactive accumulation in urine over 24-hours of mice dosed with a radioactive bolus of [^14^C]-citric acid, and treated with either vehicle or compound **2** (*P = 0.07, Student’s t test, n = 5). (**D**) Incorporation of radioactive [^14^C]-citric acid into lipids in mice treated with either vehicle or compound **2** (**P < 0.01, Student’s t-test, n = 8). (**E**) Plasma glucose concentrations in DIO mice treated with either a single 250 mg/kg dose of compound **2**, or 3 days 250 mg/kg BID with compound **2** (n = 9–10).

Steady state intravenous infusion of **2** in rat demonstrates significant uptake of the compound in the liver and kidney, as compared to the plasma while exhibiting minimal brain penetration (Figure S4). In spite of its doubly-charged and polar nature, **2** achieved significant exposures in mice following oral dosing. When treated with 250 mg/kg *po*, the unbound concentration of **2** in liver tissue was over 4 fold higher than the IC_50_ observed in mouse hepatocyte (4.5 μM, Fig. 1) for a period of 8 hours (Fig. 4A). Thus, we were able to assess the effect of NaCT inhibition on citrate uptake into a variety of tissues or matrices (liver, urine, kidney and white adipose tissue) *in vivo* (Fig. 4B,C). Using the same 250 mg/kg *po* dose of **2,** either acutely or sub-chronically (BID dosing for 3 days), labelled citrate uptake in liver was reduced by 33% relative to controls. Treatment with compound **2** increased the counts detected in urine collected over 24 hours after administration of a bolus of radioactive labeled citrate (Fig. 4C). Total kidney radioactivity increased although this is likely at least partially attributed to urine contamination (Fig. 4B). We selected white adipose tissue as one tissue with minimal *SLC13a5* expression, and as anticipated there were no differences in the radioactive counts between drug-treated and control groups. Consistent with the reduction in hepatic citrate uptake, conversion of labelled citrate into hepatic lipids was also decreased (Fig. 4D), and furthermore, lower uptake of citrate by liver in treated mice was associated with modest but significant reductions in plasma glucose concentrations (Fig. 4E).

**Figure 5:**
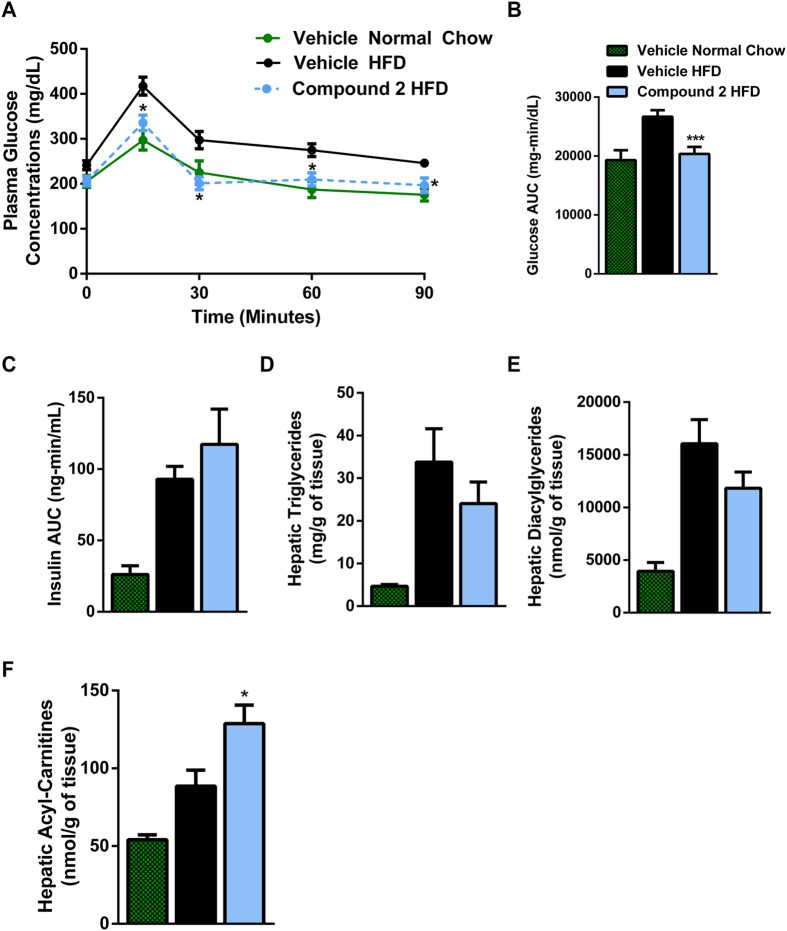
Effect in mice treated chronically with 2. (**A**) Plasma glucose concentrations during an OGTT following treatment for 20 days with 250 mg/kg BID compound **2** (*P < 0.05, n = 5 normal chow, n = 10 for each HFD group). (**B**) Plasma glucose concentrations AUC (***P < 0.005, One-way ANOVA – Dunnett’s post hoc test). (**C**) Plasma insulin concentrations AUC. (**D–F**) Hepatic triglycerides, diacylglycerides, and acyl-carnitines from livers of mice treated with 250 mg/kg BID compound **2** for 21 days (n = 5 normal chow, n = 10 for each HFD group, *P < 0.05, One-way ANOVA – Dunnett’s post hoc test).

Given these positive effects of **2** on plasma glucose concentrations, we subsequently treated mice fed a HFD for 21 days with either 250 mg/kg of **2** BID or vehicle. During an oral glucose tolerance test (OGTT), the HFD group had significant glucose intolerance compared to the normal chow fed mice. The glucose intolerance was completely reversed in the mice treated with **2** (Fig. 5A,B). During the OGTT there were no differences in plasma insulin concentrations area under the curve (Fig. 5C). In addition, livers of mice treated with **2** had a trend for lower hepatic TAG and DAG, with higher levels of acylcarnitine (Fig. 5D–F), which may be indicative of greater flux through β-oxidation pathways and was corroborated by a trend for increase β-hydroxybutyrate concentrations (Vehicle HFD: 0.40 ± 0.02 mmol/L and **2** HFD: 0.49 ± 0.06 mmol/L).

## Discussion

NaCT has been proposed as a therapeutic target with the potential for simultaneously regulating both *de novo* lipogenesis and hepatic glucose production via a single mechanism. Herein, we describe compound **2** which to our knowledge is the first potent and selective pharmacological NaCT inhibitor capable of blocking citrate uptake both *in vitro* and *in vivo*. Compound **2** was able to reduce glucose excursion during an OGTT following chronic oral administration. This reduction in glucose excursion is consistent with the positive metabolic effects observed with knock-out or knock-down of *SLC13a5* in mice and rats, respectively^19^.

A number of other known solute carrier protein inhibitors are highly similar structurally to their corresponding biological substrates. Examples include the sodium-coupled glucose transporters (SGLT1/2) for which glucose-derived inhibitors were developed^25^,^26^,^27^. Despite structural resemblance of the inhibitors to glucose, selectivity appears to be achievable within the SLC5 family. An analogous approach was adopted for the identification of the NaCT inhibitor dicarboxylate **1** which was selected by way of its structural similarity with citrate. Encouragingly, the active (*R*)-enantiomer **2** was highly selective for NaCT relative to NaDC1 and NaDC3. The activity of **2** on other known members of the SLC13 family (NaS1 and NaS2) was not tested, but is expected to be negligible as they are known to transport sulfate, not dicarboxylates or citrate^28^.

While evaluating the mode of action for **2**, it was established that the inhibitor’s interaction with NaCT is direct, specific, stereosensitive, sodium-requiring and competitive with citrate. These findings, along with the asymmetric distribution of the compound in cells expressing NaCT, support that **2** is recognized as a substrate for active cellular uptake. Accordingly, *in vitro* and *in vivo* inhibition of citrate transport appears to be a consequence of direct inhibition of NaCT by binding of **2** directly to NaCT. However, the data reported here cannot rule out potential indirect modulation of NaCT via another unidentified intracellular target.

Recently, Mancusso *et al.* published the crystalline structure of the bacterial homolog for the SLC13 family of transporters, VcINDY^29^. The transporter was isolated in its inward-facing conformation, bound to a molecule of citrate and one sodium ion. The functional characterization of VcINDY was subsequently studied by Mulligan *et al.*^30^. It is proposed that the transporter undergoes an alternating access mechanism resulting in exposure of the binding site from one side of the membrane to the other via an enclosed intermediate state. The citrate molecule was also proposed to be a state-dependent inhibitor by selectively blocking the transporter in its inward-facing intracellular conformation. In analogy with the work reported herein, compound **2** may also inhibit the transport of citrate by stabilization of the inward-facing conformation of NaCT. This hypothesis is supported by two primary findings: first, since **2** is a substrate for NaCT, it can presumably occupy the citrate binding site and second, inhibition can be achieved with the presence of **2** in the intracellular domain. While this model is consistent with all data reported herein, additional studies are needed to fully confirm this hypothesis.

Compound **2** is a low molecular weight and polar dicarboxylate which provides attributes such as excellent aqueous solubility, very low metabolic clearance and selectivity against other targets. However, the low lipophilicity and predominantly dianionic nature of **2** at physiological pH, presumably drive very low passive permeability. In spite of this, **2** achieved pharmacologically active exposures in mice when dosed orally. Because of its low permeability, it is possible that NaCT-mediated active transport contributes to the relatively high hepatic exposure of **2**. Active uptake may also play a role in gut absorption of **2** via intestinal solute carriers, such as NaDC1^31^. Due to its hydrophilicity and low molecular weight, paracellular absorption cannot be ruled out as another potential mechanism for oral absorption of **2**^32^,^33^.

Blocking citrate uptake in human hepatocytes reduced the incorporation of labeled citrate into downstream TCA cycle intermediates, lipids, and glucose which indicates a role for the cellular uptake of citrate in regulating metabolism. The contribution of plasma citrate to the intracellular citrate pool compared to the generation of citrate from the mitochondria is unknown. However, these data indicate a meaningful contribution of plasma citrate to the intracellular intermediate pool. Moreover citrate is a key signaling metabolite regulating glycolysis, and lipid metabolism by inducing polymerization and activation of ACC^9^,^10^. The effect of NaCT inhibition on glycolysis is further supported by the reduction in ECAR in the presence of physiological citrate concentrations and *in vivo* reductions in acute plasma glucose concentrations. Furthermore, in mice chronically treated with **2**, the observed reductions in glucose excursion during an OGTT can be attributed to a combination of the direct effect of NaCT inhibition and the adaptive changes in flux rates which occur upon NaCT inhibition^19^.

*In vivo* the ~33% reduction in hepatic citrate uptake in mice dosed with **2** is consistent with the reduction in hepatic citrate uptake in *SLC13a5−/−* mice^19^. The incomplete inhibition of hepatic citrate uptake in *SLC13a5−/−* mice and **2** treated mice may be due at least partially to the compensatory uptake by other citrate transporters NaDC1 and NaDC3. The contribution of NaDC1 and NaDC3 to hepatic cellular citrate transport is likely smaller in human since these genes are expressed to a lower degree relative to *SLC13A5* in human liver. The greater downstream inhibitory effect of **2** on the incorporation of citrate into lipids is also consistent with the *SLC13a5−/−* mice in which ~90% reduction incorporation into fatty acids was observed in isolated hepatocytes^19^. A recent study identifying *SLC13A5* as a downstream target of PXR shows that knockdown of *SLC13A5* by siRNA in HepG2 cells caused significant reductions in lipids as quantified using BODIPY staining of the lipids^34^. While the molecular mechanisms underlying the broader effects of inhibiting NaCT on lipid synthesis compared to direct inhibition of citrate uptake are not clear, possible factors include specific channeling of extracellularly-derived citrate into lipids, or greater depletion of a key pool of citrate than predicted by the reduction in uptake. The modest changes in hepatic lipid concentrations observed in the chronically treated mice may be due to **2** not sufficiently inhibiting NaCT throughout the dosing interval. These findings may indicate that greater coverage of the target is necessary to suppress lipid synthesis chronically. The increase in acylcarnitines would support elevated β-oxidation in mice chronically treated with **2**, consistent with the observation of increased CO_2_ production from radioactive oleate in hepatocytes isolated from the *SLC13a5−/−* mice^19^.

The significant increase in kidney radioactivity appears to be at least partially attributed to urinary contamination, since urinary radioactivity over a 24-hour collection increased for mice treated with **2**. This indicates a redistribution of plasma citrate from liver uptake to kidney uptake and/or urinary excretion in animals treated with **2**. The increase in urinary citrate with NaCT inhibition has the potential to impact kidney stone formation, as increased urinary citrate is associated with a lower risk of nephrolithiasis^35^. There were no appreciable differences in citrate uptake in the white adipose tissue which is expected considering the limited expression of *SLC13a5* in this tissue.

*SLC13A5* expression was detected in human liver, testis and neurons^36^,^37^. As reported by Thevenon *et al.*, *SLC13A5* variants were associated with epileptic encephalopathy characterized by seizures, abnormal electro-encephalogram, psychomotor delay and/or cognitive deterioration^38^. The mutation sites identified in their work were found to alter highly conserved nucleotides and amino acid residues, and were predicted as likely deleterious. The authors speculated that the mutations disrupt the ability of NaCT to bind sodium, negatively affecting the transport of citrate across the membrane. In brief, their work recognized risks associated with NaCT inhibition in neurons. Exposure of **2** was found to be restricted in the brain during the *in vivo* experiments reported herein, as expected due to the compound’s low passive permeability. Consistent with this, no phenotype analogous to epileptic encephalopathy was observed during the *in vivo* experiments, and the animals appeared in overall good condition.

In summary, we introduce here compound **2** (or PF-06649298) as the first potent and selective chemical probe suitable for inhibition of NaCT *in vitro* and *in vivo*, which was discovered using a substrate-based design strategy. Using this tool, we have demonstrated that pharmacological inhibition of NaCT recapitulates the main features previously reported for *SLC13a5−/−* animals, specifically reduction in hepatic lipid production and in plasma glucose levels following OGTT. It has proven challenging to identify novel therapeutic targets that may simultaneously reduce hepatic glucose and lipid production. For this reason, inhibition of NaCT may be an attractive therapeutic target to treat NAFLD and T2D.

## Methods

### IC_50_ in HEK_NaCT_ cells

Protocols to generate the stable cell line overexpressing NaCT or cell transfection with *SLC13A2* or *SLC13A3* can be found in the supporting information. HEK-293 cells stably transfected with the human sodium Citrate transporter SLC13A5 or transiently transfected with the human SLC13A2 or SLC13A3 transporter were removed from the flasks with dissociation buffer and re-suspended in an equal volume of growth media (DMEM, High Glucose supplemented with 10% heat inactivated fetal bovine serum and 500 μg/ml Geneticin). For the transient transfections, Geneticin was eliminated from the media. Cell suspensions were centrifuged at 1,000 rpm for approximately 5 minutes. Resulting cell pellets were re-suspended in growth media and placed in a 37 °C water bath for approximately 10 minutes prior to a final spin at 1,000 rpm. Following this spin the resulting cell pellets were re-suspended in assay buffer, 142 mM NaCl (or 142 mM choline chloride), 5 mM KCl, 1.2 mM MgSO_4_, 1.5 mM CaCl, 5 mM glucose and 25 mM HEPES pH 7.4. Cell suspensions were counted and the concentration adjusted as needed with assay buffer to 2e^6^ viable cells /mL. 20 μL of cell suspension were added to 384-well cyotstar-T plates followed by the addition of 5 μL of 10× concentration of compound. Cells and compound were incubated at 37 °C for approximately 30 minutes prior to the addition of 25 μL of 300 μM citric acid (16 μM radiolabeled [^14^C]-citric acid and 284 μM cold citric acid) to make a 150 μM final concentration of citric acid or 21 μM [^14^C]-succinic acid. After substrate addition, the uptake of radioactivity was monitored on Perkin Elmer Microbeta plate reader. Data from reads between 45–90 minutes was used to assess the ability of test compounds to inhibit the uptake of citric or succinic acid.

### Cell viability

For cell viability, cells are incubated with compound for 90 minutes at 37 ^o^C. Following this incubation, 25 μL Cell Titer-Glo (Promega) was added to the plates which were then analyzed on the Envision (luminescence).

### IC_50_ in cryopreserved human and mouse hepatocytes

Cryopreserved mouse or human hepatocytes were plated in a 48- or 96-well collagen-coated plate respectively at 50,000 cells/well in recovery/plating media (Corning 454534). The cells were allowed to attach to the plate for 3 hours in an incubator at 37 °C under 5% CO_2_. Subsequently, the media was aspirated and replaced with Williams E media supplemented with 100 nM Dexamethasone, 1× ITS+ (Corning: 354352), 1× pen/strep and 1× L-glutamine and placed in an incubator at 37 °C under 5% CO_2_ overnight. On the day of the experiment, compounds were added to the media at the appropriate concentration and incubated for 30 minutes. Following the incubation step, citrate (pH = 7.4) was added to the hepatocytes as a mixture of unlabeled (Sigma Aldrich) and 1 μCi 1,5-[^14^C]-labeled citrate (PerkinElmer NEC160250UC) resulting in the final concentration of 150 μM citrate concentration per well. For the control background wells, 100 μL of RIPA buffer was added to the cells. The cells were incubated for 40 and 165 minutes for the human and mouse hepatocytes respectively. To stop the reaction, cells were washed with phosphate buffer saline (PBS) followed by the addition of 100 μL and 50 μL of RIPA buffer for the mouse and human hepatocytes respectively. For the human hepatocytes, 45 μL of the resulting solution was added to a Perkin Elmer isoplate along with 200 μL of Optiphase Supermix (Perkin Elmer 1200-439). Subsequently, plates were analyzed by a Perkin Elmer Microbeta Trilux scintillation counter. For the mouse hepatocytes, 95 μL of the resulting solution was added to a 7 mL glass scintillation vial along with 6 mL of Optiphase Supermix. Samples were analyzed on a Beckman Tri-Carb liquid scintillation counter.

Incorporation of [^14^C]-citrate into triglycerides by human hepatocytes was measured using the same procedure as above except cells were incubated with the radioactive citrate for 24 hours. Subsequently, the media was aspirated and 100 μL of isopropyl alcohol:THF (9:1) was added. The plate was shaken for 15 minutes, centrifuged at 3000 revolutions per minute for 5 minutes, and 50 μL of supernatant from each well was applied per TLC lane (Whatman LK6D Silica Gel Plates). Radiolabeled lipids were resolved using a 2-solvent system: Solvent 1 contained a 100:100:100:40:36 mixture of ethyl acetate:isopropyl alcohol:CHCl_3_:MeOH:0.25% KCl and Solvent 2 a 70:27:3 hexane:diethyl ether:acetic acid mixture. The TLC plate was dried under nitrogen for 30 minutes and [^14^C]-standards added to a vacant lane. Bands were visualized and quantitated using a Molecular Dynamics’ Storm 860 PhosphorImager system following 18–36 hours exposure to a Phosphorimager screen.

### OCR and ECAR measurements in HEK_NaCT_ cells incubated with citrate treatment, with and without test compounds

HEK_NaCT_ cells were plated at 12,000 cells/well onto a poly-d-lysine coated XF96 plate (Sigma, Seahorse Biosciences). Cells were plated in culture media containing HG DMEM, 10% FBS, 2 mM glutamine, 1 mM pyruvate, 0.5 mg/mL Geneticin, and 1% penicillin/streptomycin (Life Technologies) and incubated overnight at 37 ^o^C. The following day, the media was changed to Seahorse Biosciences assay medium (Seahorse Biosciences) supplemented with 10 mM glucose and 2 mM pyruvate (Sigma). The plate was pre-incubated for 1 hour in a 37 °C air incubator. Thereafter, oxygen consumption rate (OCR) and extracellular acidification rate (ECAR) were measured at baseline and following injection of test compounds (30 μM), sodium citrate (1.5 mM, Sigma) using a XF96 analyzer (Seahorse Bioscience) as described^39^.

### Measurement of TCA cycle intermediates

Primary hepatocytes were given the indicated substrates (compound and labeled citrate) under the same conditions used to generate the IC_50_ curves. Incubations were stopped by rapidly removing culture media and adding 80% methanol pre-cooled in dry ice. Cellular debris was removed by centrifugation and samples dried by Speedvac. Dried cell extracts were then re-dissolved in 100 μL HPLC-grade water. Citrate uptake was analyzed by injecting a 10 μL aliquot of sample onto an Imtakt UK-Phenyl (2.1 mm × 150 mm) column (Imtakt USA, Philadelphia, PA, USA) using a Waters Acquity UPLC system (Waters Corp., Milford, MA, USA) coupled to a Thermo Q-Exactive mass spectrometer (Thermo Corp., San Jose, CA, USA). Analytes were chromatographically resolved using a linear gradient from 0% to 50%, or acetonitrile containing 0.3% formic acid, over three minutes at 500 μL min^−1^ with a column temperature of 40 °C. Metabolites were monitored using negative ion electrospray, ESI(-), in full scan mode with a resolution of 70,000 FWHM calibrated to mass accuracy <2 ppm. Instrument parameters were fixed: sheath gas 60 AU, auxiliary gas 40 AU, sweep gas 2 AU, ESI(-) spray voltage −3 kV, capillary temperature 320 °C, and S-lens set at 50 AU. Citrate transport was determined through enrichment of ^13^C_6_-citrate into the intracellular pool of citrate as determined by the percent ^13^C_6_-citrate in the total pool. Fate of citrate carbon through one turn of the TCA cycle was analyzed by comparing percent labeling into the respective pools of ^13^C_4_-fumarate, ^13^C_4_-malate, and ^13^C_4_-glutamate. Thermo LCQuan (Thermo Corp, San Jose, CA, USA) was used to extract out exact mass using a 5 ppm window at the calculated accurate mass for each metabolite and its isotopomer. Peak areas were used to calculate individual isotopomer percentages which were normalized to the total sum of all detected isotopomers after natural isotope correction. Data analysis was performed using Microsoft Excel and plots were generated in Graphpad Prism.

### *In vivo* experiments with compound 2

All procedures performed on animals minimized animal suffering, complied with regulations, were carried out in accordance with the approved guidelines and were approved by a Pfizer Institutional Animal Care and Use Committee. A jugular catheter was placed into the animals one week prior to conducting the experiment. On the day of the experiment, each C57/B6 fed a high-fat diet for the preceding 12-weeks received compound **2** at 250 mg/kg or vehicle. The sub-chronic mouse group received 4 previous 250 mg/kg BID doses on the previous 2 days at 7 AM and 4 PM. To assess uptake of citrate, animals were administered *i.v.* 10 μCi of 1,5-[^14^C]-citrate 90 minutes after the final dose of compound had been administered. Animals were bled at 2, 5, 10, and 20 minutes to measure plasma radioactivity. At 20 minutes, pentobarbital was administered via the jugular catheter and cardiac blood, liver, kidney, and WAT were rapidly collected from the animal. To collect urine, animals were placed in a urine collection cage for 24 hours.

In a satellite group of animals, livers were collected at 2, 4, 8 and 24 hours following a single dose of compound **2** at 250 mg/kg. Tissue was homogenized in 60:40 isopropanol: water (5-fold) and analyzed via LC/MS/MS to determine drug concentrations. Following assessment of tissue binding by equilibrium dialysis^40^ free drug concentration was determined.

Tissue samples weighing 100–300 mg were homogenized in H_2_O at a ratio of 1 mL/100 mg of tissue sample, and then placed in a 100 °C water bath for exactly 10 minutes. Treated samples were then centrifuged at 10,000 RPM for 30 minutes. Subsequently, 250 μL of the supernatant was added to 9 mL of ECOlite solution, and ^14^C was counted using a scintillation counter. For *in vivo* lipid determination, 300–500 mg of tissue was added to NaOH, and incubated at 60 °C until the tissue was fully degraded. A standard lipid extraction was used to quantify the incorporation of radioactive citrate into lipid^41^.

In studies requiring chronic treatment with compound, mice were purchased at 18 weeks of age following 13 weeks of HFD administration (D12459i; 60% of the calories from fat). Mice were maintained on HFD for the duration of the study in a 12:12-h light/dark cycle. They were either dosed with 250 mg/kg of compound **2** or vehicle BID for 21 days. To perform an oral glucose tolerance test (day 20 of dosing), mice were fasted at 6:00 AM to 12:00 PM and administered P.O. 1.5 g/kg at 12:00 PM. Glucose concentrations were determined using an AlphaTrak (Abbott Laboratories) at 0, 15, 30, 60, and 90 minutes. On day 21, mice were fasted from 6:00 AM, dosed at 8:00 AM with either compound **2** or vehicle, and at 12:00 PM, mice were euthanized using CO_2_, and their livers rapidly extracted and frozen in liquid N_2_. Hepatic lipids were measured by LC-MS/MS as previously described^42^.

### Clearance in human and mouse hepatocytes

Clearance in human and mouse hepatocytes were determined as described previously in Di *et al.*, (2012)^43^.

### Passive permeability

Passive permeability was assessed using MDCK-LE cell monolayer as described previously in Di *et al.*, (2011)^44^.

### Binding experiments with [^3^H]-2

Parental HEK-293 cells or HEK-293 cells expressing *SLC13A5* were incubated with [^3^H]-**2** at 75 nM. See supplemental methods for additional details.

### Intra- and extracellular concentrations of compound in cellular assays

k_puu_ values were determined by measuring total cell and media concentrations corrected for their respective fraction unbound. Detailed methods to determine intra- and extracellular concentrations of compound in the HEK_NaCT_ cells and human hepatocytes will be the subject of a separate manuscript that is under preparation.

## Additional Information

**How to cite this article:** Huard, K. *et al.* Discovery and characterization of novel inhibitors of the sodium-coupled citrate transporter (NaCT or SLC13A5). *Sci. Rep.* **5**, 17391; doi: 10.1038/srep17391 (2015).

## Supplementary Material

Supplementary Information

## Figures and Tables

**Table 1:** Inhibition of citrate uptake and asymmetric distribution of the inhibitor in the cellular assays.

Compound	HEK_NaCT_		HEK_NaDC1_	HEK_NaDC3_	Cryopreserved human hepatocytes	
	IC_50_ (μM)	K_puu_	IC_50_ (μM)	IC_50_ (μM)	IC_50_ (μM)	K_puu_
1 (Racemate)	0.80 (6.1 ± 0.4)	ND	ND	ND	ND	ND
2 (*R* enantiomer)	0.41 (6.4 ± 0.3)	29 ± 5	>100	>100	16.2 (4.8 ± 0.1)	4.8 ± 0.5
3 (*S* enantiomer)	>25	0.6 ± 0.2	ND	ND	>25	0.4 ± 0.1
4 (*R* enantiomer)	0.44 (5.3 ± 0.1)	ND	5.5^a^ (5.3 ± 0.1)	3.2^a^ (5.5 ± 0.1)	ND	ND
5 (*R* enantiomer)	>25	ND	ND	ND	6.76 (5.2 ± 0.1)	ND

HEK_NaCT_, HEK_NaDC1_ and HEK_NaDC3_ are HEK-293-derived cells overexpressing NaCT, NaDC1 and NaDC3 respectively. Unless otherwise noted, IC_50_ values are reported as a geometric mean of at least 3 replicates with pIC_50_ ± SD in parentheses. K_puu_ is the ratio of free intracellular and media concentration of inhibitor after 1 h incubation at a concentration of 1 μM. The K_puu_ values are reported as a geometric mean of 3 replicates ± SD. ND = not determined.

^a^Mean of 2 replicates.
