# Supplementary material for: Discovery and characterization of novel inhibitors of the sodium-coupled citrate transporter (NaCT or SLC13A5)
Source: Sci Rep. 2015 Dec 1;5:17391. doi: 10.1038/srep17391 (PMC4664966; doi:10.1038/srep17391)
Supplement: Supplementary Information [file srep17391-s1.pdf]

Supporting Information

Discovery and characterization of novel inhibitors of the sodium-coupled citrate transporter (NaCT or SLC13A5)

Kim Huard*, Janice Brown, Jessica C. Jones, Shawn Cabral, Kentaro Futatsugi, Matthew Gorgoglione, Adhiraj Lanba, Nicholas B. Vera, Yimin Zhu, Qingyun Yan, Yingjiang Zhou, Cecile Vernochet, Keith Riccardi, Angela Wolford, David Pirman, Mark Niosi, Gary, Aspnes, Michael Herr, Nathan E. Genung, Thomas Magee, Daniel P. Uccello, Paula Loria, Li Di, James R. Gosset, David Hepworth, Timothy Rolph, Jeffrey A. Pfefferkorn, Derek M. Erion*

Table of Contents:

Supporting Data

Figure S1: Structure and profiling of previously reported NaCT inhibitor **S1** in our assays-------S2

Figure S2: X-ray analysis of crystal structure of **3**--S3

Figure S3: Inhibition of citrate uptake with **2** in cryopreserved A) human and B) mouse hepatocytes---S3

Table S1: Selectivity data for **2**--S4

Figure S4: Free concentrations of compound **2** following intravenous infusion in rat-----------S6

Scheme S1: Synthesis of dicarboxylates **1**-**3**--S6

Scheme S2: Synthesis of dicarboxylate **4**--S7

Scheme S3: Synthesis of dicarboxylate **[^{3}H]-2**---S7

Scheme S4: Synthesis of ester **5**--S7

Supporting Experimental

Materials and Methods--S8

Synthesis and characterization for all compounds and intermediates--------------------------------S8

Generation of the stable cell line expressing *SLC13A5*---S17

Generation of cells expressing *SLC13A2* or *SLC13A3*---S17

Crystallization and X-ray analysis of compound **3**--S18

Supporting data

Figure S1. Structure and profiling of previously reported NaCT inhibitor **S1** in our assays. A) Structure of previously reported NaCT inhibitor (**S1**).(Pajor and Randolph, 2007) B) Inhibition of citrate uptake in HEK_{NaCT} cells and C) cell viability in CellTiter-Glo® assay for compound **S1**.

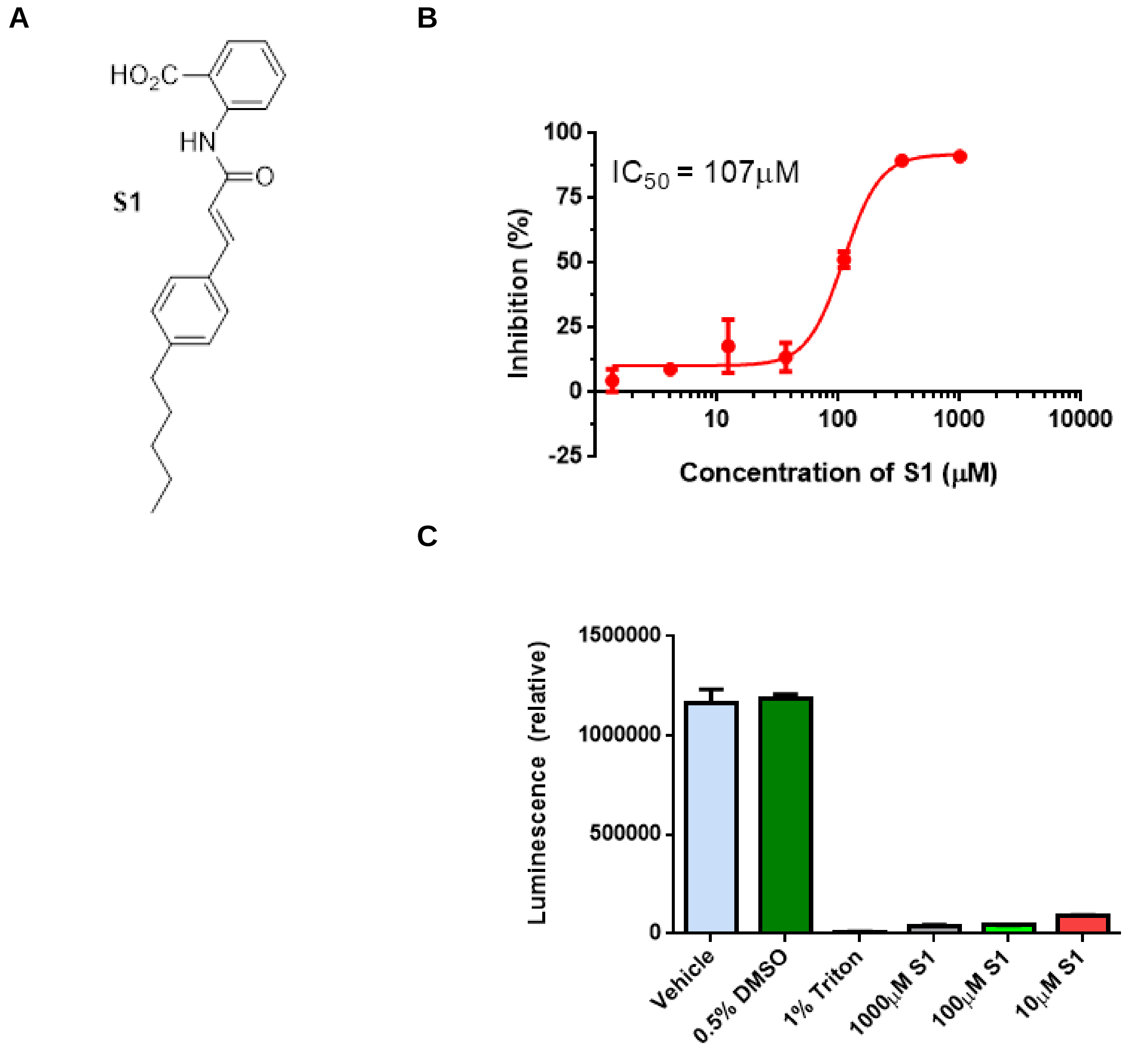

Figure S2. X-ray analysis of crystal structure of **3**. Asymmetric unit of **3** with displacement parameters drawn at 50% probability

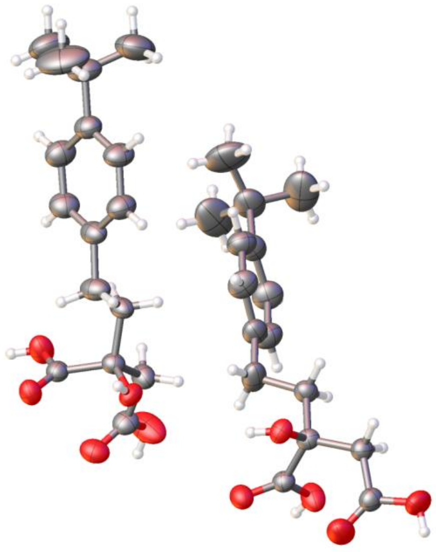

Figure S3. Inhibition of citrate uptake with **2** in cryopreserved A) human and B) mouse hepatocytes

A **B**

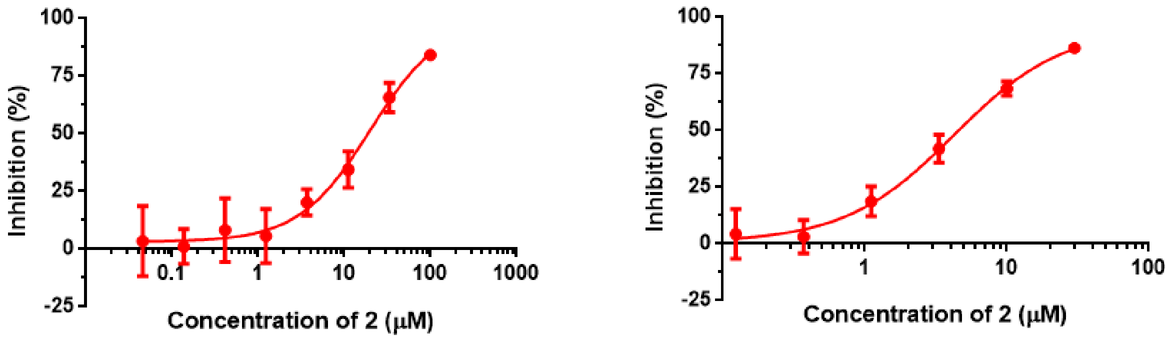

Table S1. Selectivity data for **2**

Targets	% inhibition			IC$_{50}$ (µM)
	3 µM	10 µM	100 µM	
**Enzymes**				
Abelson murine leukemia viral oncogene homolog 1 (ABL1)			<10	
Angiotensin-converting enzyme 1 (ACE1)			<10	
Acetylcholinesterase (ACHE)			<10	
Cyclooxygenase 2 (prostaglandin endoperoxide synthase 2)			22	
Cytochrome P450 1A2 (CYP1A2)	<10			
Cytochrome P450 2C8 (CYP2C8)	<10			
Cytochrome P450 2C9 (CYP2C9)	<10			
Cytochrome P450 2D6 (CYP2D6)	<10			
Cytochrome P450 3A4 (CYP3A4)	12			
Diacylglycerol acyltransferase 1 (DGAT1)				>50
Diacylglycerol acyltransferase 2 (DGAT2)				>50
Lymphocyte-specific protein tyrosine kinase (LCK)			<10	
Monoamine oxidase A (MAOA)			<10	
Monoacylglycerol acyltransferase 1 (MGAT1)				>50
Monoacylglycerol acyltransferase 2 (MGAT2)				>50
Monoacylglycerol acyltransferase 3 (MGAT3)				>50
Mitogen-activated protein kinase 14 (MAPK14 / P38-alpha)			<10	
Phosphodiesterase 3A1 (PDE3A1)				>30
Phosphodiesterase 3B (PDE3B)			<10	
Phosphodiesterase 4D (PDE4D)			<10	
Phosphodiesterase 4D3 (PDE4D3)				>30
Phosphodiesterase 5A1 (PDE5A1)				>30
Proto-oncogene tyrosine-protein kinase (SRC)			<10	
**Transporters / ion channels**				
ATP-binding cassette, sub-family B, member 11 (ABCB11)				>200
Calcium channel, L-type				>30
Choline transporter (CHT1) (SLC5A7)			<10	
Dopamine transporter, sodium-dependent (DAT)			<10	
Human ether-a-go-go (hERG)				>100
Noradrenaline transporter, sodium-dependent (NAT)			<10	
Potassium voltage-gated channel, KQT-like subfamily, member 1(KCNQ1)		<10		
Serotonin transporter, sodium-dependent (5-HTT)			<10	
Sodium channel, neuronal alpha-subunit (NaV1.1)		<10		
Sodium channel, type V, alpha-subunit (NaV1.5)		<10		
**Receptors**				
Adenosine receptor A1 (ADORA1)			<10	
Adrenergic receptor, alpha-1A (ADRA1A)			<10	
Adrenergic receptor, alpha-2A (ADRA2A)			<10	

Adrenergic receptor, alpha-2B (ADRA2B)			<10	
Adrenergic receptor, beta-1 (ADRB1)			<10	
Adrenergic receptor, beta-2(ADRB2)			<10	
Androgen receptor			<10	
Angiotensin II receptor type 1 (AGTR1)			<10	
Arginine vasopressin receptor 1A (AVPR1A)			<10	
Cannabinoid receptor 1 (CB1)			<10	
Cholecystokinin receptor B (CCKBR)			<10	
Dopamine receptor 1 (D1)			<10	
Dopamine receptor 2 (D2)			<10	
Endothelin (ETA)			<10	
Epidermal growth factor receptor (EGFR)			<10	
Gamma-aminobutyric acid receptor A, alpha 1 (GABRA1)			<10	
Glucocorticoid receptor (GR)			<10	
Histamine receptor 1 (H1)			<10	
Histamine receptor 2 (H2)			<10	
Histamine receptor (H3)			<10	
Muscarinic acetylcholine receptor 1 (M1)			<10	
Muscarinic acetylcholine receptor 2 (M2)			<10	
Muscarinic acetylcholine receptor 3 (M3)			<10	
Neurokinin receptor 1 (NK1)			<10	
Nicotinic acetylcholine receptor complex (alpha-4 / beta-2)			<10	
Opioid receptor, kappa 1 (OPRK1)			13	
Opioid receptor, mu (MOP)			<10	
Peroxisome proliferator activated receptor gamma (PPAR-gamma)			<10	
Serotonin receptor 1A (5-HT1A)			<10	
Serotonin receptor 1B (5-HT1B)			<10	
Serotonin receptor 2A (5-HT2A)			<10	
Serotonin receptor 2B (5-HT2B)			<10	
Serotonin receptor 3 (5-HT3)			<10	
Serotonin receptor 4e (5-HT4e)			<10	
Vascular endothelial growth factor receptor 2 (VEGFR-2)			<10	

Figure S4. Free concentrations of compound **2** following intravenous infusion (0.27mg/kg/hour) in rat

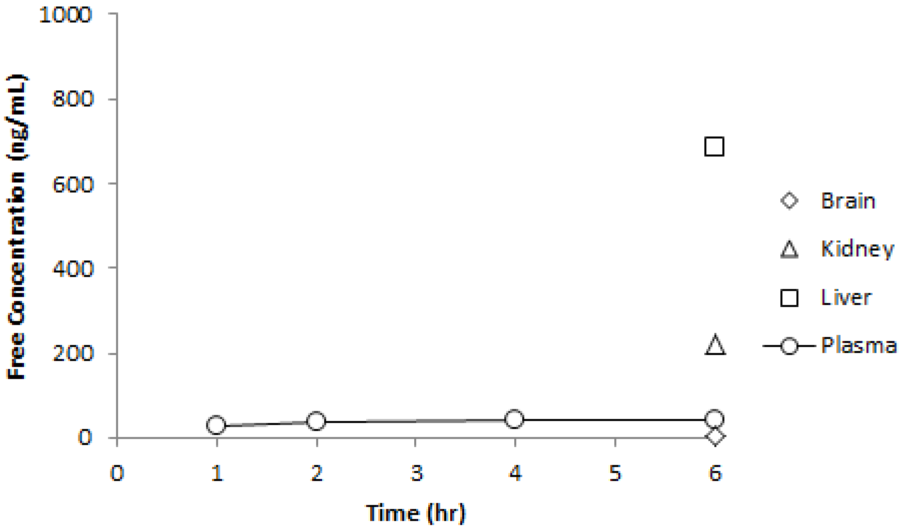

Scheme S1. Synthesis of dicarboxylates **1-3**

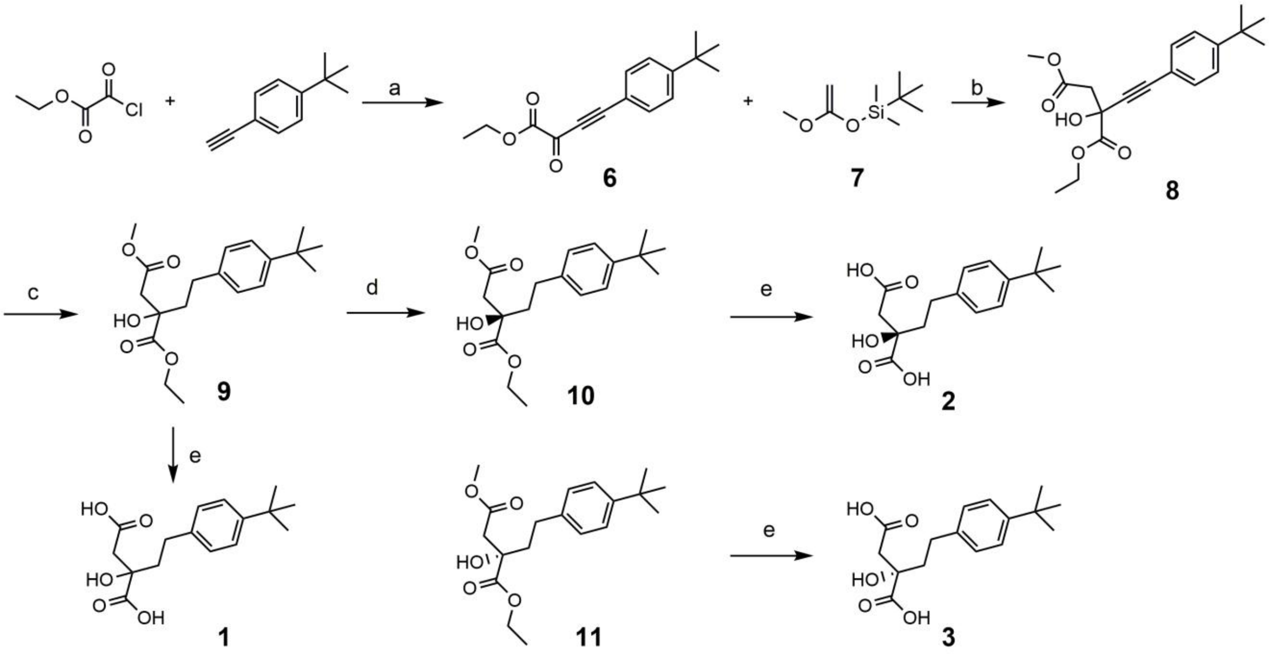

(a) 15 mol% CuI, TEA, THF, 16 h, 71%. (b) 28 mol% $CuCl_2$, THF, 16 h, 67%. (c) 10% Pd/C w/v, 45 psi H_2, EtOH, 1 h, 95%. (d) chiral separation. (e) NaOH, THF, 85-99%.

Scheme S2. Synthesis of dicarboxylate **4**

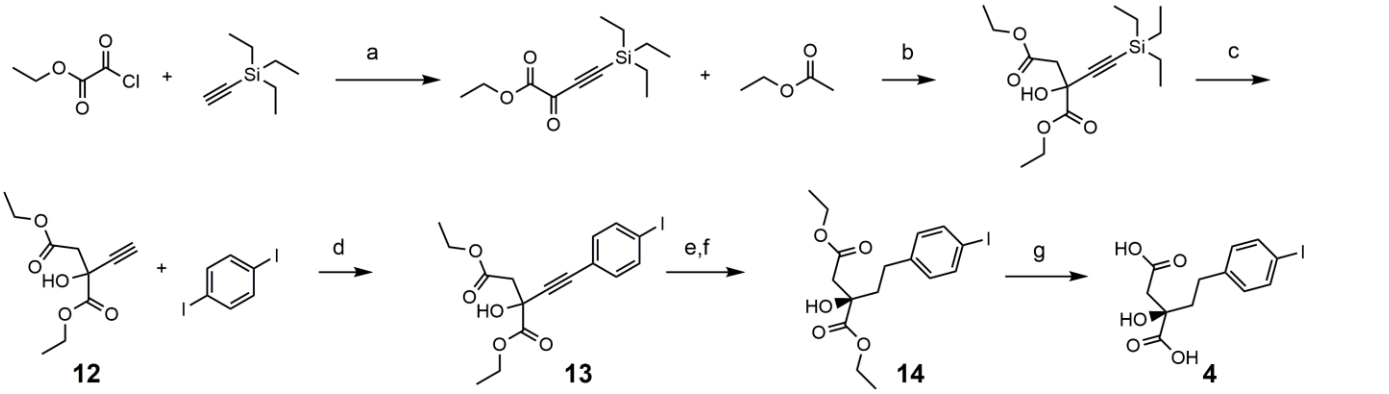

(a) 5 mol% CuI, TEA, THF, 16 h, 91%. (b) LiHMDS, THF, 5 h, 44%. (c) TBAF, THF, 1 h, 71%. (d) 20 mol% CuI, 20 mol% TPP, piperidine, 10 mol% $PdCl_2(ACN)_2$, THF, 0.5 h, 37%. (e) p-tolylhydrazide, xylenes, 8 h, 55%. (f) chiral separation. (g) NaOH, THF, 54%.

Scheme S3. Synthesis of dicarboxylate **[^{3}H]-2**

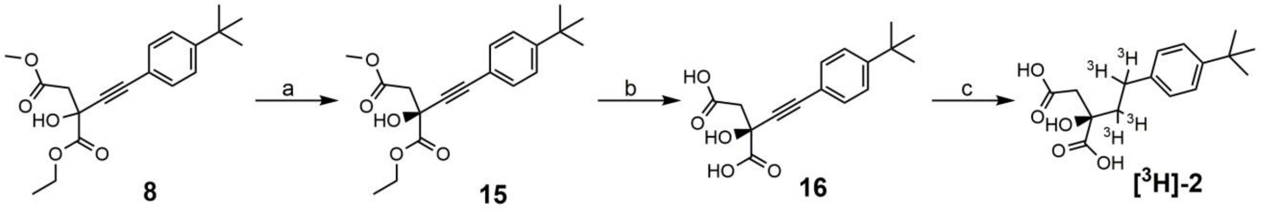

(a) chiral separation. (b) NaOH, rt, 16 h, 91%. (c) $^3H_2$, 3:1 EtOH:water, Pd/C.

Scheme S4. Synthesis of ester **5**

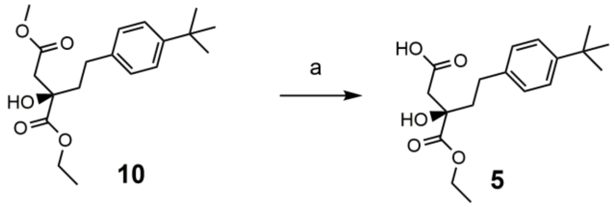

(a) NaOH, EtOH, 30 min, 66%.

Supporting Experimental

Materials and Methods. All chemicals, reagents, and solvents were purchased from commercial sources and used without further purification unless otherwise noted. ^{1}H NMR spectra were recorded with 400 or 500 MHz spectrometers and are reported relative to residual undeuterated solvent signals. Data for ^{1}H NMR spectra are reported as follows: chemical shift (δ, ppm), multiplicity, coupling constant (Hz), and integration. The multiplicities are denoted as follows: s, singlet; d, doublet; t, triplet; q, quartet; spt, septet; m, multiplet; br s, broad singlet. Liquid chromatography mass spectrometry (LC-MS) was performed on an Agilent 1100 Series (Waters Atlantis C18 column, 4.6 x 50 mm, 5 μm; 95% water/acetonitrile linear gradient to 5% water/acetonitrile over 4 min, hold at 5% water/ 95% acetonitrile to 5.0 min, trifluoroacetic acid modifier (0.05%); flow rate of 2.0 mL/ min). Silica gel chromatography was performed using a medium pressure Biotage or ISCO system and columns prepackaged by various commercial vendors including Biotage and ISCO. Preparative high-performance liquid chromatography (HPLC) purification was performed on a Waters FractionLynx preparative chromatography system using a Phenomenex Gemini/Synergi C18 5μm 150 x 21.2 mm 5μm column and eluting with water and acetonitrile with (0.1% TFA, formic acid, or NH_4OH) at 30 mL/min for 10 minutes. All tested compounds were determined to be >95% pure by HPLC by UV peak detection at 215 nm. The terms "concentrated" and "evaporated" refer to the removal of solvent at reduced pressure on a rotary evaporator with a water bath temperature not exceeding 60 °C. All procedures and experiments involving animals were carried as per the protocols and guidelines reviewed and approved by Pfizer Institutional Animal Care and Use Committee

Synthesis and characterization for all compounds and intermediates

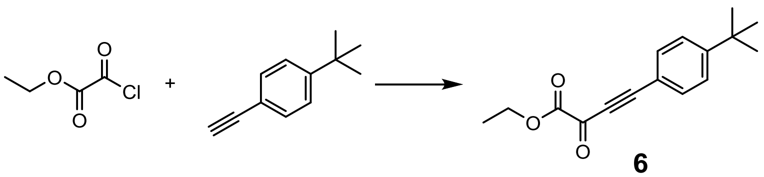

6

Ethyl 4-(4-(tert-butyl)phenyl)-2-oxobut-3-ynoate (6). Ethyl chlorooxoacetate (70.6 mL, 632 mmol) was added via an addition funnel to a yellow solution of 1-tert-butylethynylbenzene (58 mL, 320 mmol), copper iodide (3.0 g, 15.8 mmol), and triethylamine (90 mL, 650 mmol) in tetrahydrofuran (600 mL) in a 3-neck, 3 L round-bottom flask equipped with a thermocouple and overhead stirrer. The internal temperature was controlled between 21 and 24 ^{o}C with an ice bath during the addition. The yellow solution turned to a hazy, orange suspension. The reaction was

stirred at room temperature for 16 h. Ethyl chlorooxoacetate (15 mL, 134 mmol) and copper(I) iodide (850 mg, 4.5 mmol) in tetrahydrofuran (50 mL) were added to the reaction mixture and stirred at room temperature for 4 h. Copper(I) iodide (2.60 g, 13.7 mmol) in tetrahydrofuran (50 mL) was added to the reaction mixture at room temperature and stirred for 24 h. Copper(I) iodide (1.8 g, 9.4 mmol) was added in tetrahydrofuran (100 mL) to the reaction mixture at room temperature and stirred for 48 h. A saturated aqueous solution of sodium bicarbonate (300 mL) was added to the reaction mixture and stirred at room temperature for 15 minutes. The suspension turned to a biphasic solution which was extracted with ethyl acetate (3x 300 mL). The combined organics were washed with brine (200 mL), dried over magnesium sulfate, filtered, and concentrated to dryness. The residue was purified by flash column chromatography (10-25% EtOAc in Heptanes) to afford ethyl 4-(4-(tert-butyl)phenyl)-2-oxobut-3-ynoate (**6**) (58 g, 71%) as an orange oil. ^{1}H NMR (500 MHz, $CDCl_3$) δ 1.33-1.35 (m, 9H), 1.43 (t, *J*=7.6 Hz, 3H), 4.42 (q, *J*=7.1 Hz, 2H), 7.45 (d, *J*=8.5 Hz, 2H), 7.62 (d, *J*=8.3 Hz, 2H).

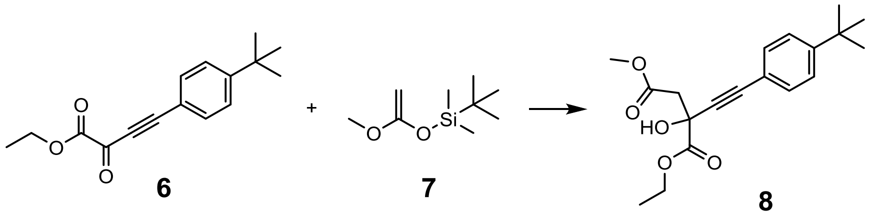

6 7 8

1-Ethyl 4-methyl 2-((4-(tert-butyl)phenyl)ethynyl)-2-hydroxysuccinate (8). Copper(II) chloride (5.2 g, 37 mmol) was added to an orange solution of ethyl 4-(4-(tert-butyl)phenyl)-2-oxobut-3-ynoate (**6**) (34.3 g, 132 mmol) in tetrahydrofuran (350 mL) at room temperature. The reaction mixture was stirred at room temperature for 50 minutes. 1-(tert-butyldimethylsilyloxy)-1-methoxyethene (**7**) (35.1 mL, 150 mmol) was added. The cloudy, orange suspension turned to a clear, green solution and a mild exotherm was observed. The reaction was stirred at room temperature for 5 hours. 1-(tert-butyldimethylsilyloxy)-1-methoxyethene (**7**) (6 mL, 27.5 mmol) was added and stirring was continued at room temperature for 16 hours. The reaction mixture was cooled in an ice bath and saturated ammonium chloride (200 mL) was added. The biphasic, pale green solution was stirred for 30 minutes and then extracted with ethyl acetate (3x 200 mL). The combined organics were washed with brine (100 mL), dried over magnesium sulfate, concentrated to dryness. The residue was purified by flash column chromatography (20-30% EtOAc in Heptanes) to afford 1-ethyl 4-methyl 2-((4-(tert-butyl)phenyl)ethynyl)-2-hydroxysuccinate (**8**) (30 g, 67%) as an orange oil. ^{1}H NMR (500 MHz, $CDCl_3$) δ 1.31 (s, 9H),

1.35 (t, J=7.3 Hz, 3H), 3.15 (d, J=16.3 Hz, 1H), 3.28 (d, J=16.6 Hz, 1H), 3.71-3.73 (m, 3H), 4.34-4.41 (m, 2H), 7.33 (d, J=8.5 Hz, 2H), 7.37 (t, J=8.3 Hz, 2H).

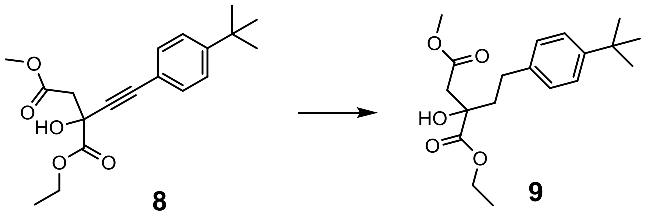

1-Ethyl 4-methyl 2-(4-(tert-butyl)phenethyl)-2-hydroxysuccinate (9). Ethanol (50 mL) was added to palladium on carbon (4.0 g) in a hydrogenation vial. 4-ethyl 1-methyl 2-((4-(tert-butyl)phenyl)ethynyl)-2-hydroxysuccinate (**8**) (37.2 g, 112 mmol) in ethanol (300 mL) was added to the suspension. The reaction was shook at room temperature under 45 psi of hydrogen for 1 h. The suspension was filtered over celite and the solid washed with ethanol (30 mL). The filtrate was concentrated and dried under high vacuum to yield 1-ethyl 4-methyl 2-(4-(tert-butyl)phenethyl)-2-hydroxysuccinate (**9**) (36 g, 95%) as an orange oil. ^{1}H NMR (500 MHz, $CDCl_3$) δ 1.30-1.37 (m, 12H), 1.96-2.11 (m, 2H), 2.46-2.53 (m, 1H), 2.73-2.85 (m, 2H), 2.98 (d, J=16.4 Hz, 1H), 3.71 (s, 3H), 4.21-4.32 (m, 2H), 7.13 (d, J=7.47 Hz, 2H), 7.31 (d, J=7.47 Hz, 2H); m/z = 358.9 $[M+Na]^+$.

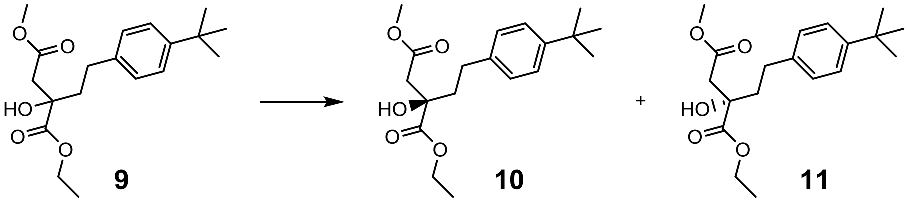

Chiral separation of 1-Ethyl 4-methyl 2-(4-(tert-butyl)phenethyl)-2-hydroxysuccinate (9). Enantiomers of 4-ethyl 1-methyl 2-(4-(tert-butyl)phenethyl)-2-hydroxysuccinate (**9**) (42g, 125 mmol) were separated by SFC purification on a 30x 250 mm Chiralpak AD-H column using 85:15 CO_2/methanol at 120 g/minute with monitoring at 210 nm.

1-Ethyl 4-methyl (R)-2-(4-(tert-butyl)phenethyl)-2-hydroxysuccinate (10). 1-Ethyl 4-methyl (*R*)-2-(4-(tert-butyl)phenethyl)-2-hydroxysuccinate (**10**) eluted at 8.31 minutes to yield 16.7 g (40%) as a red oil. ^{1}H NMR (500 MHz, $CDCl_3$) δ 1.30-1.37 (m, 12H), 1.96-2.11 (m, 2H), 2.46-2.53 (m, 1H), 2.73-2.85 (m, 2H), 2.98 (d, J=16.4 Hz, 1H), 3.71 (s, 3H), 4.21-4.32 (m, 2H), 7.13 (d, J=7.5 Hz, 2H), 7.31 (d, J=7.5 Hz, 2H); $[\alpha]_D$ = +14.7° (c 0.0127, MeCN).

1-Ethyl 4-methyl (S)-2-(4-(tert-butyl)phenethyl)-2-hydroxysuccinate (11). 1-Ethyl 4-methyl (*S*)-2-(4-(tert-butyl)phenethyl)-2-hydroxysuccinate (**11**) eluted at 6.05 minutes to yield 19.3 g (46%) as a brown oil. ^{1}H NMR (500 MHz, $CDCl_3$) δ 1.30-1.37 (m, 12H), 1.96-2.11 (m, 2H), 2.46-2.53 (m, 1H), 2.73-2.85 (m, 2H), 2.98 (d, *J*=16.4 Hz, 1H), 3.71 (s, 3H), 4.21-4.32 (m, 2H), 7.13 (d, *J*=7.5 Hz, 2H), 7.31 (d, *J*=7.5 Hz, 2H); $[\alpha]_D$ = -15.8° (*c* 0.0203, MeCN).

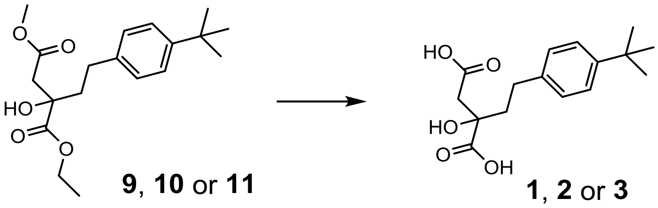

General hydrolysis protocol. Diester **9**, **10** or **11** (1.0 g, 2.9 mmol) was dissolved in THF (10 mL). 1N sodium hydroxide (11.8 mL, 11.8 mmol) was added at room temperature. The biphasic mixture was stirred at room temperature for 16 h. Water (50 mL) was added to the reaction mixture which was extracted with a 1:2 mixture of ethyl acetate/heptane (50 mL). The aqueous layer was acidified to a pH of 3 with 1N hydrogen chloride (12 mL). The aqueous layer was extracted with ethyl acetate (2x 100 mL). The organic layer was dried over magnesium sulfate, filtered and concentrated to give an off-white solid. The material was recrystallized in methyl *t*-butyl ether/heptane (3 mL/30 mL) to yield dicarboxylate **1**, **2** or **3** as a white solid (775-905 mg, 85-99%).

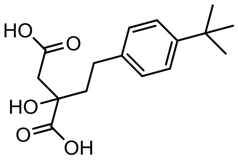

2-(4-(tert-butyl)phenethyl)-2-hydroxysuccinic acid (1). ^{1}H NMR (500 MHz, $CDCl_3$) δ 1.31 (s, 9H), 1.99-2.14 (m, 2H), 2.57 (td, *J*=12.9, 5.0 Hz, 1H), 2.78-2.88 (m, 2H), 3.08 (d, *J*=17.1 Hz, 1H), 7.13 (m, *J*=8.3 Hz, 2H), 7.31 (m, *J*=8.3 Hz, 2H).

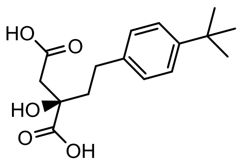

(R)-2-(4-(tert-butyl)phenethyl)-2-hydroxysuccinic acid (2). ^{1}H NMR (500 MHz, $CDCl_3$) δ 1.31 (s, 9H), 2.00-2.15 (m, 2H), 2.52-2.61 (m, 1H), 2.79-2.87 (m, 2H), 3.07 (d, *J*=16.6 Hz, 1H), 7.13 (d, *J*=7.5 Hz, 2H), 7.31 (d, *J*=7.5 Hz 2H); $[\alpha]_D$ = +7.7° (*c* 0.0238, $CHCl_3$); m/z = 293.4 $[M-H]^-$.

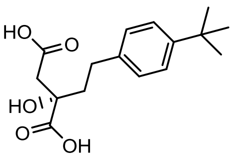

(S)-2-(4-(tert-butyl)phenethyl)-2-hydroxysuccinic acid (3). ^{1}H NMR (500 MHz, $CDCl_3$) δ 1.31 (s, 9H), 2.00-2.15 (m, 2H), 2.52-2.61 (m, 1H), 2.79-2.87 (m, 2H), 3.07 (d, *J*=16.6 Hz, 1H), 7.13 (d, *J*=7.5 Hz, 2H), 7.31 (d, *J*=7.5 Hz, 2H); $[\alpha]_D$ = -6.05° (*c* 0.0245, $CHCl_3$); m/z = 293.4 $[M-H]^-$.

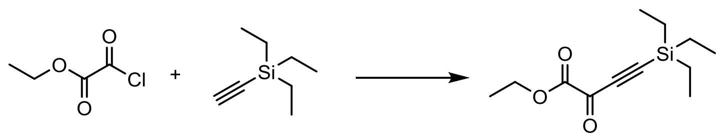

Ethyl 2-oxo-4-(triethylsilyl)but-3-ynoate . Triethyl(ethynyl)silane (4.0 mL, 22 mmol) and ethyl 2-chloro-2-oxoacetate (4.0 mL, 36 mmol) were added to a clear solution of triethylamine (5.3 mL, 38 mmol) and copper iodide (206 mg, 1.1 mmol) in tetrahydrofuran (70 mL) at room temperature. The reaction mixture was stirred at room temperature for 16 h. Saturated sodium carbonate (100 mL) was added and the reaction mixture was extracted with ethyl acetate (3x 50 mL). The combined organics were dried over sodium sulfate and concentrated to dryness. The residue was purified by flash column chromatography (0-20% ethyl acetate in heptanes) to afford ethyl 2-oxo-4-(triethylsilyl)but-3-ynoate (4.74 g, 91%) as a pale yellow oil. ^{1}H NMR (400 MHz, $CDCl_3$) δ 0.63 (q, *J*=7.4 Hz, 6H), 0.95 (t, *J*=8.2 Hz, 9H), 1.29 (t, *J*=7.2 Hz, 3H), 4.27 (q, *J*=7.3 Hz, 2H).

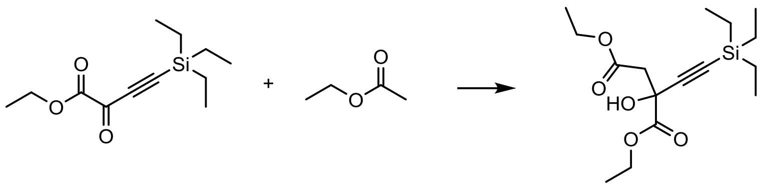

Diethyl 2-hydroxy-2-((triethylsilyl)ethynyl)succinate. Ethyl acetate (1.63 ml, 16.5 mmol) was added to a solution of 1M LiHMDS (16.5 ml, 16.5 mmol) in tetrahydrofuran (20.0 ml) at -78 ^{o}C. The pale yellow reaction mixture was stirred at -78 ^{o}C for 30 minutes. 2-Oxo-4-triethylsilanyl-but-3-ynoic acid ethyl ester (2.5 g, 10.4 mmol) in tetrahydrofuran (20 ml) was added at the same temperature. The reaction mixture was allowed to stir for 1 h at -78 ^{o}C, quenched with saturated ammonium chloride (20 mL) at -78 ^{o}C and extracted with ethyl acetate (2x 40 mL) to afford a yellow oil. The crude product was purified by flash column chromatography using 5% ethyl acetate in hexanes to afford diethyl 2-hydroxy-2-((triethylsilyl)ethynyl)succinate (1.5 g, 44%) as a colorless sticky liquid. ^{1}H NMR (400 MHz, $CDCl_3$) δ 0.55-0.61 (m, 6H), 0.95 (t, *J*=7.9 Hz, 9H), 1.25 (t, *J*=7.2, 3H), 1.31 (t, *J*=7.1, 3H), 3.01 (d, *J*=16.6 Hz, 1H), 3.16 (d, *J*=16.5 Hz, 1H), 4.00 (s, 1H), 4.13 (q, *J*=7.1 Hz, 2H), 4.25-4.40 (m, 2H).

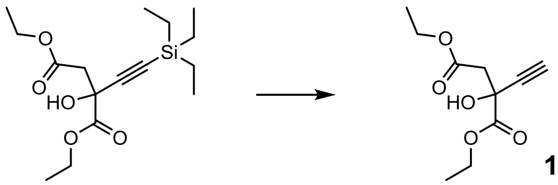
12

Diethyl 2-ethynyl-2-hydroxysuccinate (12). To a stirred solution of 2-hydroxy-2-triethylsilanylethynyl-succinic acid diethyl ester (1.5 g, 4.6 mmol) in diethyl ether was added tetrabutylammonium fluoride (6.85 mL, 6.85 mmol) at 0 ^{o}C and stirred at 0 ^{o}C for 1 h. The reaction was quenched with saturated ammonium chloride (15 mL) and extracted with diethyl ether (3x 30 mL). The combined organic layer were washed with water (25 mL) and brine (30 mL), dried over sodium sulfate, and concentrated. The crude product was purified by flash column chromatography using 10% ethyl acetate in hexanes to afford diethyl 2-ethynyl-2-hydroxysuccinate (700 mg, 71.4%) as colorless gum. ^{1}H NMR (400 MHz, $CDCl_3$) δ 1.24 (t, *J*=7.1, 3H), 1.33 (t, *J*=7.1, 3H), 2.53 (s, 1H), 3.01 (d, *J*=16.5 Hz, 1H), 3.15 (d, *J*=16.5 Hz, 1H), 4.06 (s, 1H), 4.14 (q, *J*=7.1 Hz, 2H), 4.28-4.38 (m, 2H).

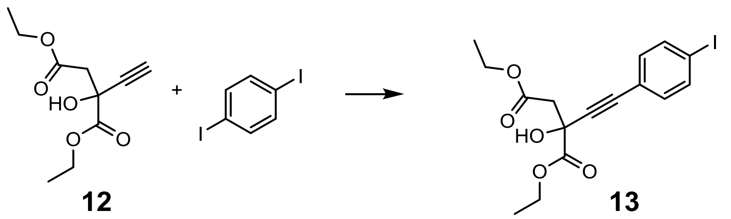

12 13

Diethyl 2-hydroxy-2-((4-iodophenyl)ethynyl)succinate (13). Diethyl 2-ethynyl-2-hydroxysuccinate (**12**) (350 mg, 1.63 mmol) and 1,4-diiodo-benzene (1.62 g, 4.90 mmol) in tetrahydrofuran (5.4 mL) was degassed with argon gas for 50 min. Triphenyl phosphine (85 mg, 0.33 mmol), Copper iodide (62 mg, 0.33 mmol), dichloro bis(acetonitrile)palladium(II) (42 mg, 0.16 mmol) and piperidine (5.4 mL) were added successively to the reaction at room temperature and stirred for 40 minutes. The reaction mixture was diluted with ethyl acetate (40 mL) and washed with 1N hydrochloric acid (3x 25 mL). The organic layer was washed with water (25 mL) and brine (30 mL), then dried over anhydrous sodium sulfate, filtered and concentrated under reduced pressure. The crude product was purified by flash column chromatography using 10% ethyl acetate in hexanes to afford diethyl 2-hydroxy-2-((4-iodophenyl)ethynyl)succinate (**13**) (250 mg, 36.8%) as brown liquid. ^{1}H NMR (400 MHz, $CDCl_3$) δ 1.26 (t, *J*=7.1, 3H), 1.34 (t, *J*=7.1, 3H), 2.53 (s, 1H), 3.09 (d, *J*=16.5 Hz, 1H), 3.22 (d, *J*=16.5 Hz, 1H), 4.10-4.20 (m, 2H), 4.30-4.40 (m, 2H), 7.14 (d, *J*=8.2 Hz, 2H), 7.64 (d, *J*=8.2 Hz, 2H).

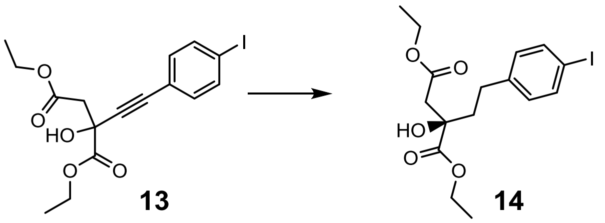

2-Hydroxy-2-[2-(4-iodo-phenyl)-ethyl]-succinic acid diethyl ester (14). *p*-Toluene-sulfonhydrazide (1.12 g, 6.01 mmol) was added to a stirred solution of 2-hydroxy-2-(4-iodo-phenylethynyl)-succinic acid diethyl ester (**13**) (500 mg, 1.20 mmol) in xylenes (25 mL). The reaction was refluxed for 8 h then cooled to room temperature and diluted with ethyl acetate (50 mL). The organics were washed with 1N sodium hydroxide (3x 20 mL) followed by brine (30 mL), dried over sodium sulfate, filtered and concentrated in vacuo. The crude material was purified by flash column chromatography using 20% ethyl acetate in hexanes as eluent to afford 2-hydroxy-2-[2-(4-iodo-phenyl)-ethyl]-succinic acid diethyl ester (**14**) (280 mg, 55%) as sticky liquid.

The enantiomers were separated by SFC purification on a 30x 250 mm Chiralpak AD-H column using 85:15 CO_2/methanol at 120g /minute with monitoring at 210 nm to afford (S)-2-Hydroxy-2-[2-(4-iodo-phenyl)-ethyl]-succinic acid diethyl ester (90 mg,) and (R)-2-Hydroxy-2-[2-(4-iodo-phenyl)-ethyl]-succinic acid diethyl ester (**14**) (91 mg) as colorless sticky solids. ^{1}H NMR (400 MHz, $CDCl_3$) δ 1.22 (t, *J*=7.2, 3H), 1.29 (t, *J*=7.1, 3H), 1.92-1.98 (m, 2H), 2.37-2.45 (m, 1H), 2.69 (d, *J*=16.2 Hz, 1H), 2.69-2.77 (m, 1H), 2.92 (d, *J*=16.2 Hz, 1H), 3.80 (s, 1H), 4.30 (q, *J*=14.3 Hz, 2H), 4.21-4.27 (m, 2H), 6.90 (d, *J*=8.2 Hz, 2H), 7.58 (d, *J*=8.2 Hz, 2H).

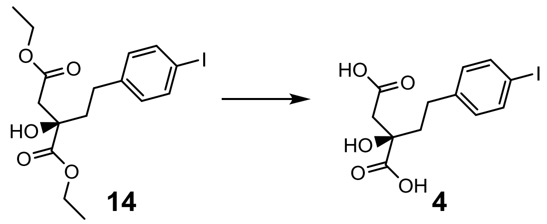

14 **4**

(R)-2-hydroxy-2-(4-iodophenethyl)succinic acid (4). 1M Sodium hydroxide (28.5 mg, 0.71 mmol) was added to a solution of (*S*)-2-Hydroxy-2-[2-(4-iodo-phenyl)-ethyl]-succinic acid diethyl ester (**14**) (75 mg, 0.18 mmol) in tetrahydrofuran (2 mL) at 5 °C. The reaction mixture was stirred at room temperature for 16 h. Water (5 ml) was added and extracted with ethyl acetate (2x 15ml). The aqueous layer was acidified with 1N hydrochloric acid to a pH of 2 and extracted with dichloromethane (2x 15ml). The combined organics layer were washed with water (20 mL) and brine (15 mL) then dried over sodium sulfate. The organic filtrate was concentrated to afford (*R*)-2-Hydroxy-2-[2-(4-iodo-phenyl)-ethyl]-succinic acid (**4**) (35 mg, 54%) as white solid. ^{1}H NMR (400 MHz, $CDCl_3$) δ 1.89-2.03 (m, 2H), 2.48 (td, *J*=13.0, 4.9, 1H), 2.66 (d, *J*=16.1 Hz, 1H), 2.76 (td, *J*=13.0, 4.9, 1H), 2.95 (d, *J*=16.1 Hz, 1H), 6.98 (d, *J*=8.3 Hz, 2H), 7.58 (d, *J*=8.2 Hz, 2H).

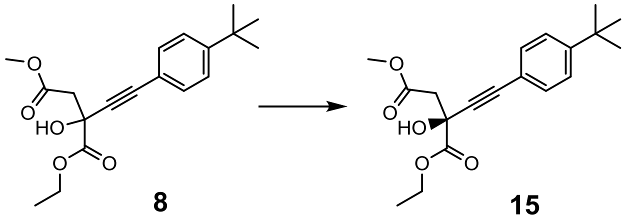

8 **15**

1-ethyl 4-methyl (S)-2-((4-(tert-butyl)phenyl)ethynyl)-2-hydroxysuccinate (15). Enantiomers of 1-Ethyl 4-methyl 2-((4-(tert-butyl)phenyl)ethynyl)-2-hydroxysuccinate (**8**) were separated by HPLC purification on a 4.6x 150 mm chiralcel-OJ-RH column using a gradient of 5% to 90% acetonitrile in water containing 0.1% trifluoroacetic acid at a flow rate of 1.0 mL/min for 18 minutes with monitoring at 210 and 254 nm. 1-Ethyl 4-methyl (R)-2-((4-(tert-butyl)phenyl)ethynyl)-2-hydroxysuccinate eluted at 12.72 minutes. 1-Ethyl 4-methyl (S)-2-((4-

(tert-butyl)phenyl)ethynyl)-2-hydroxysuccinate (**15**) eluted at 12.00 minutes and was isolated as a white solid. ^{1}H NMR (500 MHz, $CDCl_3$) δ 1.31 (s, 9H), 1.36 (t, *J*=7.1 Hz, 3H), 3.15 (d, *J*=16.3 Hz, 1H), 3.28 (d, *J*=16.3 Hz, 1H), 3.72 (s, 3H), 4.34-4.38 (m, 1H), 4.38-4.41 (m, 1H), 7.31-7.35 (m, 2H), 7.35-7.40 (m, 2H).

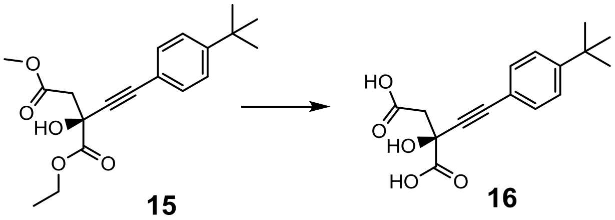

(S)-2-((4-(tert-butyl)phenyl)ethynyl)-2-hydroxysuccinic acid (16). 1-Ethyl 4-methyl (S)-2-((4-(tert-butyl)phenyl)ethynyl)-2-hydroxysuccinate (**15**) (140 mg, 0.421 mmol) was dissolved in THF (5 mL) and 1N aqueous sodium hydroxide (1.3 mL, 1.3 mmol) was added at room temperature. The reaction mixture was stirred at room temperature for 16 h. The tetrahydrofuran was concentrated under a stream of nitrogen to yield a yellow solution. Using 1N hydrogen chloride (1.5 mL), the pH of the solution was adjusted to ~4, which caused the solution to turn to a pale, tan suspension. The solid was filtered and dried under high vacuum to yield (S)-2-((4-(tert-butyl)phenyl)ethynyl)-2-hydroxysuccinic acid (**16**) (99 mg, 91%) as a tan solid. ^{1}H NMR (500 MHz, D_4-MeOH) δ 1.31 (s, 9H), 3.00 (d, *J*=16.1 Hz, 1H), 3.21 (d, *J*=16.1 Hz, 1H), 7.34-7.38 (m, 2H), 7.38-7.41 (m, 2H); $[\alpha]_D$ = 14.3° (*c* 0.0103, MeOH); m/z = 289.3 $[M-H]^-$.

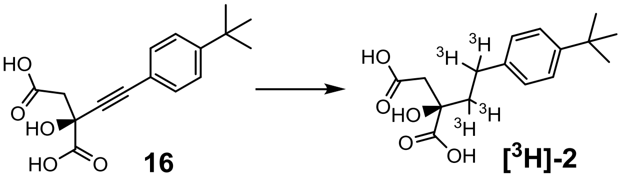

(R)-2-(2-(4-(tert-butyl)phenyl)ethyl-1,1,2,2-t4)-2-hydroxysuccinic acid ([^{3}H]-2). (S)-2-((4-(tert-butyl)phenyl)ethynyl)-2-hydroxysuccinic acid (**16**) (10 mg, 34 µM) was dissolved in a 3:1 mixture of ethanol and water (1.2 mL) and added to a tritiation vial containing a stirring bar and 10% Pd/C (2 mg). The reaction vessel was pressurized with tritium gas (40 PSI) at room temperature and left to stir for 16 h. The reaction mixture was filtered to remove the catalyst and ethanol was used to wash the solids. The crude material was purified via prep HPLC on a Phenomenex Luna Semi-prep C18 column using a gradient of 5% to 100% acetonitrile in water containing 0.2% trifluoroacetic acid at a flow rate of 2.0 mL/min for 1 h with monitoring at 254 nm. (R)-2-(2-(4-(tert-butyl)phenyl)ethyl-1,1,2,2-t4)-2-hydroxysuccinic acid (**[^{3}H]-2**) eluted at ~39 minutes. The final product was analysed by reverse phase HPLC with UV detection at 254 nm and in-line radioactivity detection on a 4.6x250 mm, 5

µm Brownlee C18 column using a gradient of 20% to 90% acetonitrile in water containing 0.2% trifluoroacetic acid over 13 minutes. The radiochemical purity was determined to be 97.1%. The specific activity was determined by mass spectral analysis to be 93.1 Ci/mmol. The radiochemical concentration of the final ethanol solution was determined to be 1 mCi/mL. The chemical identity was confirmed by coelution with the reference standard (**2**) on HPLC and by MS.

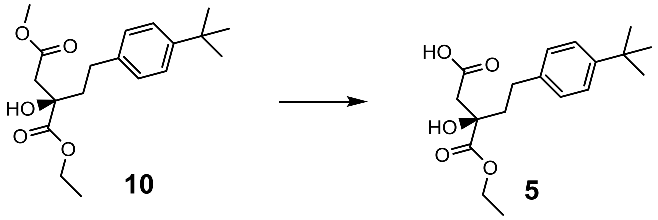

(R)-5-(4-(tert-butyl)phenyl)-3-(ethoxycarbonyl)-3-hydroxypentanoic acid (5). 1-ethyl 4-methyl (*R*)-2-(4-(tert-butyl)phenethyl)-2-hydroxysuccinate (**10**) (0.33 g, 0.98 mmol) was dissolved in ethanol (5 mL) and sodium hydroxide (39 mg, 0.98 mmol) was added while stirring at room temperature. The reaction mixture was brought to 80 °C and stirred for 30 minutes. The reaction was then cooled to room temperature and ethyl acetate (50 mL) was added. The mixture was washed with a 1M aqueous solution of sodium bisulfate (30 mL). The organic layer was washed with brine, dried over sodium sulfate and concentrated in vacuo. The crude material was purified by flash column chromatography using a gradient of 50-100% ethyl acetate in heptane as eluent to afford (*R*)-5-(4-(tert-butyl)phenyl)-3-(ethoxycarbonyl)-3-hydroxypentanoic acid (**5**) (210 mg, 66%) as a white solid. ^{1}H NMR (500 MHz, $CDCl_3$) δ 1.29-1.32 (m, 3H), 1.33 (s, 9H), 2.00-2.10 (m, 2H), 2.44-2.52 (m, 1H), 2.76-2.84 (m, 2H), 2.99-3.05 (m, 1H) 4.20-4.32 (m, 2H), 7.11-7.14 (m, 2H), 7.31-7.36 (m, 2H), 10.29-10.59 (m, 1H); $[\alpha]_D$ = 9.0° (*c* 0.0101, MeOH); m/z = 321.2 $[M-H]^-$.

Generation of the stable cell line expressing *SLC13A5*

Clones of *SLC13A5* were obtained from Origene. Isolated colonies from transfected HEK-293 cells were generated using G418 selection. Expression was confirmed by sequencing.

Generation of cells expressing *SLC13A2* or *SLC13A3*

Clones were obtained from Origene for *SLC13a2*, and *SLC13a3*. HEK-293 cells were pelleted and re-suspended in growth media with 1mM sodium butyrate at $1e^6$ viable cells per mL. For each transfection, 1.75 mL of Opti-MEM was added per 35µg of cDNA and 122.5 µL of Fuegene HD to a clean tube. This mixture was mixed and allowed to incubate at room temperature for 30 minutes. After the incubation, this mixture was added to the cells in 19 mL of media. These cells

were plated, and incubated for 48 hours. Following the incubation, NaDC1 and NaDC3 assays were executed identical to the procedure for NaCT.

Binding experiments with [^{3}H]-2. Parental HEK-293 cells or cells overexpressing *SLC13A5* were incubated in the media with 75 nM of [^{3}H]-**2**. These cells were also incubated with either 0, 20, 75, 150, 400, 750, 1,500, or 5,000 μM of pH = 7.4 unlabeled citric acid. The uptake of [^{3}H]-**2** was monitored for 2-hours. The uptake of radioactivity was monitored on Perkin Elmer Microbeta plate reader. In experiments in which cold **2** was added to the media, after 2-hours, the cells were washed 3x with PBS, and then in fresh media 100 μM cold **2** was added for 2-hours and the disappearance of radioactivity was monitored using a Perkin Elmer Microbeta plate reader.

Intra- and extracellular concentrations of compound in cellular assays. k_{puu} values were determined by measuring total cell and media concentrations corrected for their respective fraction unbound. Detailed methods to determine intra- and extracellular concentrations of compound

<u>Crystallization and X-ray analysis of (S)-2-(4-(tert-butyl)phenethyl)-2-hydroxysuccinic acid (3)</u>

Crystallization conditions. Ethyl acetate (50 mL) was added to (*S*)-2-(4-(tert-butyl)phenethyl)-2-hydroxysuccinic acid (**3**) (16.7 g, 46.2 mmol). The suspension turned to a solution upon heating. The solution was heated to reflux until the total volume was reduced to ~25 mL. Hexane (25 mL) was added and the mixture was brought back to reflux than allowed to slowly cool to room temperature. After standing at room temperature for 16 h, the resulting suspension was filtered and dried under high vacuum to yield (*S*)-2-(4-(tert-butyl)phenethyl)-2-hydroxysuccinic acid (**3**) as a white solid. The filtrate was allowed to stand at room temperature for 4 days. The solvent evaporated to ~15 mL and large, colorless crystals formed.

X-ray analysis. Data collection was performed on a Bruker APEX diffractometer at room temperature. Data collection consisted of 3 omega scans and low angle and three at high angle; each with 0.5 step. In addition, 2 phi scans were collected to improve the quality of the absorption correction.

The structure was solved by direct methods using SHELX software suite in the space group $P2_1$. The structure was subsequently refined by the full-matrix least squares method. All non-hydrogen atoms were found and refined using anisotropic displacement parameters.

During refinement, a disordered hexane resolved to anisotropic refinement, although some of these assignments were tentative. The lack of hydrogen bonding group near this region suggest that this moiety is non-polar. The SQUEEZE routine was used and this disordered solvent was removed. The SQUEEZE details are provided within the .cif file. The molecular formula given is based off of a half occupied solvent molecule.

Not all of the OH protons could be found from the difference map; these protons bonded to oxygen were placed in calculated positions. The remaining hydrogen atoms were placed in calculated positions and were allowed to ride on their carrier atoms. The final refinement included isotropic displacement parameters for all hydrogen atoms.

Analysis of the absolute structure using likelihood methods (Hooft 2008) was performed using PLATON (Spek 2010). The results indicate that the absolute structure has been correctly assigned. If the assumption is made that the compound is enantiopure, the method calculates that the probability that the structure is correct is 100.0. The Hooft parameter is reported as -0.02 with an esd of 0.13. Note that the Hooft parameter was found to be in good agreement both before and after SQUEEZE routine.

The final R-index was 5.8%. A final difference Fourier revealed no missing or misplaced electron density.

Pertinent crystal, data collection and refinement are summarized in Table S2. Atomic coordinates, bond lengths, bond angles, Torsion angles and displacement parameters are listed in Tables S3–S6.

Table S2. Crystal data and structure refinement for z222.

Identification code	z222_sq_d
Empirical formula	C19 H29 O5
Formula weight	337.42
Temperature	296(2) K
Wavelength	1.54178 Å
Crystal system	Monoclinic
Space group	P2(1)

Unit cell dimensions	a = 10.8003(3) Å	□= 90°.
	b = 10.6845(3) Å	□= 96.074(2)°.
	c = 16.8514(5) Å	□ = 90°.
Volume	1933.66(10) $Å^3$	
Z	4	
Density (calculated)	1.159 Mg/m^3	
Absorption coefficient	0.672 mm^{-1}	
F(000)	732	
Crystal size	0.56 x 0.21 x 0.21 mm^3	
Theta range for data collection	2.64 to 68.56°.	
Index ranges	-11<=h<=12, -12<=k<=12, -20<=l<=20	
Reflections collected	24177	
Independent reflections	6643 [R(int) = 0.0723]	
Completeness to theta = 68.56°	94.6 %	
Absorption correction	Empirical	
Max. and min. transmission	0.8718 and 0.7048	
Refinement method	Full-matrix least-squares on F^2	
Data / restraints / parameters	6643 / 1 / 391	
Goodness-of-fit on F^2	1.000	
Final R indices [I>2sigma(I)]	R1 = 0.0583, wR2 = 0.1554	
R indices (all data)	R1 = 0.0639, wR2 = 0.1600	
Absolute structure parameter	0.05(19)	
Largest diff. peak and hole	0.238 and -0.264 e.$Å^{-3}$	

Table S3. Atomic coordinates (x 10^4) and equivalent isotropic displacement parameters ($Å^2$x 10^3)
for z222. U(eq) is defined as one third of the trace of the orthogonalized U^{ij} tensor.

	x	y	z	U(eq)
C(1)	499(5)	10303(6)	4844(2)	104(2)
C(2)	-750(7)	11382(6)	5825(4)	133(2)
C(3)	-1286(5)	9272(6)	5329(3)	108(2)
C(4)	-199(3)	10151(3)	5558(2)	68(1)
C(5)	683(3)	9651(3)	6259(2)	53(1)
C(6)	1726(3)	10328(3)	6543(2)	60(1)
C(7)	2516(3)	9937(3)	7200(2)	59(1)
C(8)	2296(3)	8830(3)	7584(2)	50(1)
C(9)	1286(3)	8134(3)	7291(2)	59(1)
C(10)	475(3)	8536(3)	6653(2)	62(1)
C(11)	3134(3)	8414(3)	8310(2)	58(1)
C(12)	3905(3)	7254(2)	8141(1)	44(1)
C(13)	4520(2)	6577(2)	8885(1)	37(1)
C(14)	3485(2)	6137(2)	9370(1)	38(1)
C(15)	5280(3)	5477(2)	8621(2)	48(1)
C(16)	5559(3)	4475(2)	9239(2)	47(1)
C(17)	5847(5)	7136(7)	4968(3)	116(2)
C(18)	4437(6)	6139(6)	5863(3)	115(2)
C(19)	3888(6)	8164(7)	5274(3)	121(2)
C(20)	4979(4)	7348(4)	5608(2)	73(1)
C(21)	5710(3)	7966(3)	6342(2)	55(1)
C(22)	6673(3)	7324(3)	6786(2)	61(1)
C(23)	7280(3)	7827(3)	7469(2)	61(1)
C(24)	6990(3)	9005(3)	7738(2)	51(1)
C(25)	6084(3)	9654(3)	7275(2)	60(1)
C(26)	5455(3)	9151(3)	6598(2)	61(1)
C(27)	7642(3)	9517(4)	8499(2)	65(1)
C(28)	8919(2)	10043(2)	8392(1)	42(1)

C(29)	9665(2)	10552(2)	9164(1)	35(1)
C(30)	9754(2)	9487(2)	9772(1)	37(1)
C(31)	10923(2)	11021(2)	8969(1)	40(1)
C(32)	11831(2)	11329(2)	9676(2)	44(1)
O(1)	5346(2)	7379(2)	9353(1)	43(1)
O(2)	3257(2)	6623(2)	9987(1)	44(1)
O(3)	2824(2)	5242(2)	9011(1)	54(1)
O(4)	6049(3)	3480(2)	8941(2)	82(1)
O(5)	5344(2)	4566(2)	9921(1)	52(1)
O(6)	9036(2)	11594(1)	9468(1)	39(1)
O(7)	10545(2)	8619(2)	9593(1)	54(1)
O(8)	9123(2)	9402(2)	10320(1)	47(1)
O(9)	11681(2)	11136(3)	10350(1)	70(1)
O(10)	12846(2)	11856(2)	9440(1)	63(1)

Table S4.   Bond lengths [Å] and angles [°] for z222.

C(1)-C(4)	1.494(7)	C(29)-O(6)	1.427(3)
C(2)-C(4)	1.531(7)	C(29)-C(31)	1.518(3)
C(3)-C(4)	1.521(6)	C(29)-C(30)	1.527(3)
C(4)-C(5)	1.533(4)	C(30)-O(8)	1.209(3)
C(5)-C(6)	1.381(4)	C(30)-O(7)	1.317(3)
C(5)-C(10)	1.393(5)	C(31)-C(32)	1.499(3)
C(6)-C(7)	1.389(4)	C(32)-O(9)	1.181(3)
C(7)-C(8)	1.380(4)	C(32)-O(10)	1.331(3)
C(8)-C(9)	1.369(4)		
C(8)-C(11)	1.510(4)	C(1)-C(4)-C(3)	107.7(4)
C(9)-C(10)	1.382(4)	C(1)-C(4)-C(2)	112.9(5)
C(11)-C(12)	1.537(4)	C(3)-C(4)-C(2)	107.0(4)
C(12)-C(13)	1.537(3)	C(1)-C(4)-C(5)	109.4(3)
C(13)-O(1)	1.415(3)	C(3)-C(4)-C(5)	112.3(3)
C(13)-C(14)	1.527(3)	C(2)-C(4)-C(5)	107.6(3)
C(13)-C(15)	1.527(3)	C(6)-C(5)-C(10)	116.7(3)
C(14)-O(2)	1.211(3)	C(6)-C(5)-C(4)	120.1(3)
C(14)-O(3)	1.304(3)	C(10)-C(5)-C(4)	123.2(3)
C(15)-C(16)	1.501(3)	C(5)-C(6)-C(7)	121.9(3)
C(16)-O(5)	1.200(3)	C(8)-C(7)-C(6)	120.6(3)
C(16)-O(4)	1.311(3)	C(9)-C(8)-C(7)	117.8(3)
C(17)-C(20)	1.518(6)	C(9)-C(8)-C(11)	121.2(3)
C(18)-C(20)	1.500(6)	C(7)-C(8)-C(11)	120.9(3)
C(19)-C(20)	1.524(7)	C(8)-C(9)-C(10)	121.8(3)
C(20)-C(21)	1.543(4)	C(9)-C(10)-C(5)	121.1(3)
C(21)-C(26)	1.374(5)	C(8)-C(11)-C(12)	112.2(2)
C(21)-C(22)	1.396(5)	C(11)-C(12)-C(13)	115.1(2)
C(22)-C(23)	1.373(4)	O(1)-C(13)-C(14)	110.04(18)
C(23)-C(24)	1.385(5)	O(1)-C(13)-C(15)	107.5(2)
C(24)-C(25)	1.373(4)	C(14)-C(13)-C(15)	111.45(19)
C(24)-C(27)	1.499(4)	O(1)-C(13)-C(12)	111.22(19)
C(25)-C(26)	1.374(4)	C(14)-C(13)-C(12)	107.73(19)
C(27)-C(28)	1.517(4)	C(15)-C(13)-C(12)	108.89(19)
C(28)-C(29)	1.554(3)	O(2)-C(14)-O(3)	124.2(2)

O(2)-C(14)-C(13)	123.7(2)
O(3)-C(14)-C(13)	111.92(19)
C(16)-C(15)-C(13)	114.9(2)
O(5)-C(16)-O(4)	124.0(2)
O(5)-C(16)-C(15)	124.2(2)
O(4)-C(16)-C(15)	111.8(2)
C(18)-C(20)-C(17)	111.3(5)
C(18)-C(20)-C(19)	106.8(4)
C(17)-C(20)-C(19)	109.5(4)
C(18)-C(20)-C(21)	108.7(3)
C(17)-C(20)-C(21)	109.3(3)
C(19)-C(20)-C(21)	111.2(3)
C(26)-C(21)-C(22)	116.5(3)
C(26)-C(21)-C(20)	122.9(3)
C(22)-C(21)-C(20)	120.5(3)
C(23)-C(22)-C(21)	121.3(3)
C(22)-C(23)-C(24)	121.7(3)
C(25)-C(24)-C(23)	116.4(3)
C(25)-C(24)-C(27)	122.9(3)
C(23)-C(24)-C(27)	120.6(3)
C(24)-C(25)-C(26)	122.3(3)
C(25)-C(26)-C(21)	121.6(3)
C(24)-C(27)-C(28)	112.6(2)
C(27)-C(28)-C(29)	115.2(2)
O(6)-C(29)-C(31)	106.94(18)
O(6)-C(29)-C(30)	109.81(18)
C(31)-C(29)-C(30)	113.38(18)
O(6)-C(29)-C(28)	110.47(18)
C(31)-C(29)-C(28)	109.31(19)
C(30)-C(29)-C(28)	106.95(18)
O(8)-C(30)-O(7)	123.6(2)
O(8)-C(30)-C(29)	124.6(2)
O(7)-C(30)-C(29)	111.64(19)
C(32)-C(31)-C(29)	115.20(19)
O(9)-C(32)-O(10)	124.3(2)
O(9)-C(32)-C(31)	125.5(2)
O(10)-C(32)-C(31)	110.2(2)

Symmetry transformations used to generate equivalent atoms:

Table S5. Anisotropic displacement parameters ($Å^2 x 10^3$) for z222. The anisotropic displacement factor exponent takes the form: $-2\pi^2[h^2 a^{*2}U^{11} + ... + 2 h k a^* b^* U^{12}]$

	U^{11}	U^{22}	U^{33}	U^{23}	U^{13}	U^{12}
C(1)	113(4)	127(4)	66(2)	21(2)	-19(2)	0(3)
C(2)	141(5)	106(4)	138(4)	-1(3)	-57(4)	59(4)
C(3)	80(3)	125(4)	108(3)	19(3)	-37(3)	-10(3)
C(4)	70(2)	65(2)	64(2)	4(2)	-18(2)	11(2)
C(5)	51(2)	57(2)	50(1)	1(1)	-2(1)	9(1)
C(6)	62(2)	51(2)	64(2)	14(1)	-9(1)	2(1)
C(7)	54(2)	55(2)	66(2)	7(1)	-6(1)	1(1)
C(8)	54(2)	52(2)	44(1)	4(1)	5(1)	18(1)
C(9)	66(2)	47(1)	62(2)	8(1)	-1(1)	6(1)
C(10)	59(2)	56(2)	69(2)	0(1)	-7(1)	-3(1)
C(11)	64(2)	60(2)	46(1)	0(1)	-2(1)	22(1)
C(12)	48(2)	46(1)	38(1)	8(1)	6(1)	13(1)
C(13)	42(1)	32(1)	37(1)	3(1)	1(1)	1(1)
C(14)	38(1)	36(1)	39(1)	5(1)	2(1)	2(1)
C(15)	51(2)	45(1)	47(1)	6(1)	11(1)	10(1)
C(16)	49(2)	34(1)	57(1)	6(1)	2(1)	5(1)
C(17)	107(4)	165(5)	78(2)	-60(3)	12(2)	-40(3)
C(18)	125(4)	123(4)	93(3)	-18(3)	-5(3)	-76(3)
C(19)	105(4)	144(5)	99(3)	-32(3)	-48(3)	-3(3)
C(20)	77(2)	86(2)	54(2)	-18(2)	-5(2)	-26(2)
C(21)	57(2)	58(2)	48(1)	-7(1)	2(1)	-17(1)
C(22)	68(2)	51(2)	63(2)	-14(1)	-2(1)	-6(1)
C(23)	53(2)	61(2)	67(2)	2(1)	-4(1)	-2(1)
C(24)	47(2)	59(2)	47(1)	-6(1)	0(1)	-15(1)
C(25)	60(2)	52(2)	68(2)	-11(1)	-2(1)	-4(1)
C(26)	57(2)	64(2)	58(2)	1(1)	-8(1)	-5(1)
C(27)	49(2)	91(2)	54(2)	-19(2)	6(1)	-23(2)
C(28)	44(1)	43(1)	38(1)	-2(1)	1(1)	-3(1)
C(29)	38(1)	28(1)	39(1)	-2(1)	0(1)	2(1)

C(30)	38(1)	34(1)	39(1)	0(1)	2(1)	4(1)
C(31)	39(1)	38(1)	41(1)	1(1)	4(1)	-3(1)
C(32)	40(1)	42(1)	49(1)	2(1)	2(1)	-4(1)
O(1)	33(1)	40(1)	55(1)	6(1)	1(1)	1(1)
O(2)	48(1)	43(1)	42(1)	-3(1)	10(1)	-6(1)
O(3)	59(1)	56(1)	47(1)	-10(1)	10(1)	-18(1)
O(4)	124(2)	55(1)	71(1)	14(1)	25(1)	45(1)
O(5)	68(1)	40(1)	48(1)	4(1)	1(1)	7(1)
O(6)	35(1)	30(1)	53(1)	-4(1)	5(1)	0(1)
O(7)	63(1)	39(1)	63(1)	12(1)	23(1)	15(1)
O(8)	48(1)	46(1)	47(1)	7(1)	10(1)	7(1)
O(9)	58(1)	101(2)	50(1)	2(1)	-4(1)	-21(1)
O(10)	39(1)	84(2)	63(1)	13(1)	-6(1)	-16(1)

Table S6. Hydrogen coordinates (x 10^4) and isotropic displacement parameters ($Å^2$x 10^3) for z222.

	x	y	z	U(eq)
H(03C)	905	9529	4740	156
H(03D)	1112	10950	4945	156
H(03E)	-70	10525	4390	156
H(03F)	-1285	11734	5390	200
H(03G)	-89	11958	5986	200
H(03H)	-1222	11225	6266	200
H(03I)	-976	8482	5164	162
H(03J)	-1823	9631	4898	162
H(03K)	-1744	9148	5780	162
H(031)	1905	11067	6286	72
H(028)	3200	10425	7383	71
H(024)	1140	7369	7529	71
H(036)	-220	8054	6483	75
H(01E)	2629	8225	8738	69
H(01F)	3692	9093	8487	69
H(01A)	4551	7506	7816	53
H(01B)	3367	6666	7831	53
H(01C)	4831	5101	8152	57
H(01D)	6062	5795	8468	57
H(1A)	5376	6840	4490	175
H(1B)	6464	6526	5151	175
H(1C)	6249	7909	4859	175
H(3A)	3876	6304	6256	173
H(3B)	5095	5601	6086	173
H(3C)	3992	5739	5408	173
H(2A)	3287	8208	5654	181
H(2B)	3508	7806	4785	181
H(2C)	4183	8990	5173	181

H(034)	6909	6540	6615	74
H(032)	7902	7364	7759	73
H(027)	5889	10462	7425	73
H(023)	4842	9622	6307	73
H(03A)	7731	8856	8896	78
H(03B)	7135	10172	8698	78
H(02A)	8818	10716	8004	50
H(02B)	9406	9392	8172	50
H(02C)	11291	10389	8654	47
H(02D)	10795	11765	8640	47
H(1)	4980	8022	9454	64
H(3)	2267	5040	9281	80
H(4)	6204	2958	9294	124
H(6)	8435	11344	9683	59
H(7)	10408	7968	9827	81
H(10)	13395	11881	9815	94

Supplemental References

Pajor, A.M., and Randolph, K.M. (2007). Inhibition of the Na+/dicarboxylate cotransporter by anthranilic acid derivatives. Mol Pharmacol *72*, 1330-1336.
